# Suppressing Polysulfide Crossover in Sodium Polysulfide Redox-Flow Batteries with an Oxyanion-Functionalized Glass Fiber Separator

**DOI:** 10.1021/acsnano.6c04702

**Published:** 2026-06-27

**Authors:** Jieun Kang, Cheng-Tien Hsieh, Wenda Wu, Guang Yang, Nian Liu

**Affiliations:** † School of Chemical and Biomolecular Engineering, 1372 Georgia Institute of Technology, Atlanta, Georgia 30332, United States; ‡ Chemical Sciences Division, 6146 Oak Ridge National Laboratory, Oak Ridge, Tennessee 37831, United States

**Keywords:** Polymer membrane, Dip-coating, Polysulfide shuttling, Sodium−sulfur batteries, Flow batteries

## Abstract

Sodium polysulfide nonaqueous redox-flow batteries are promising candidates for grid-scale energy storage due to their high theoretical energy density and earth-abundant components. However, their performance is fundamentally limited by severe polysulfide shuttling and unstable sodium–metal interfaces, particularly under high-concentration catholyte conditions. Here, we report a scalable and perfluoroalkyl-substances-free membrane modification strategy via a simple dip-coating method to fabricate commercial glass fiber (GF) with poly­(4-styrenesulfonic acid-*co*-maleic acid) sodium salt (PSSMA). The sulfonate and carboxylate functional groups in PSSMA provide both electrostatic and steric repulsion against polysulfide anions, while preserving the mechanical flexibility of the GF substrate. The optimized PSSMA–GF membrane significantly suppresses shuttle current, mitigates self-discharge, and delays the formation of short-chain, less soluble polysulfide species, leading to improved Coulombic efficiency (from <60 to 98.3%) and stable cycling over 100 cycles in sodium polysulfide coin-type full cells. Furthermore, the coating promotes uniform solid electrolyte interphase formation on the sodium anode. The feasibility of the PSSMA–GF membrane was further demonstrated using the flow-cell configuration, showing a smooth discharge plateau at 1.5 V under 1.0 mA cm^–2^. This work demonstrates an environmentally benign, cost-effective, and easily scalable membrane fabrication strategy, offering a practical pathway to overcome key challenges in sodium polysulfide redox-flow batteries and other advanced energy storage systems.

## Graphical abstract

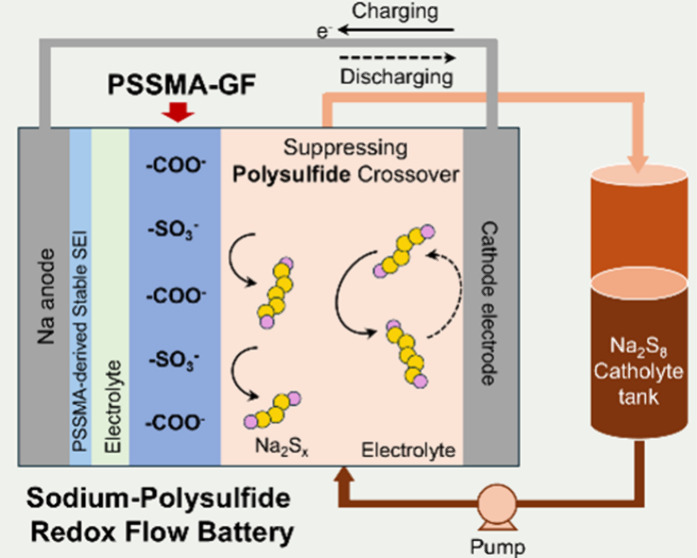

Redox-flow batteries (RFBs) have garnered significant attention as a promising platform for large-scale grid energy storage, owing to their unique architecture that decouples energy capacity from power output.^1,2^ While conventional aqueous systems such as all-vanadium and zinc–bromine redox couples have been actively explored, their limited voltage window and low energy density hinder broader implementation, while the toxicity of vanadium and bromine compounds raises additional concerns.^3,4^ As a result, nonaqueous systems have emerged as an attractive alternative due to their broader electrochemical stability window and higher operating voltages. Among them, sodium–sulfur-based nonaqueous RFBs (NARFBs) are particularly appealing due to the high theoretical capacities of both sodium and sulfur, coupled with multielectron redox reactions of polysulfides that afford open-circuit voltages (OCVs) exceeding 2.2 V.^5,6^ Recent reports have demonstrated practical discharge capacities over 7 Ah L^–1^, highlighting the potential of sodium–sulfur (Na–S) NARFBs for next-generation high-energy storage. However, the widespread application of this system is fundamentally hindered by two major challenges: the crossover of polysulfide species (Na_2_S_*x*_) and the instability of the sodium-metal anode.^7^ In flow configurations, where the catholyte must maintain high concentrations of soluble polysulfides, these issues are exacerbated. The migration of polysulfide species leads to severe shuttle effects, resulting in active material loss, insoluble Na_2_S formation on Na metal anode, and rapid degradation of Coulombic efficiency (CE) and cycle life. Additionally, the highly reactive sodium anode forms unstable solid electrolyte interphases (SEIs), causing localized current hotspots and dendrite growth that further compromise long-term cycling stability.^8^

To mitigate these problems, several membrane strategies have been investigated. For example, ceramic membranes such as Na^+^-β″-Al_2_O_3_ can physically block polysulfide transport, but their brittleness and millimeter-scale thickness limit their use in flexible or scalable flow systems.^9^ Perfluoroalkyl substances (PFAS)-based membranes like Nafion offer ionic selectivity and shuttle suppression but raise serious concerns related to sodium-metal compatibility, high cost, and environmental toxicity.^10–12^ Recent efforts to address polysulfide migration have focused on advanced functional coatings for separators, including conductive nanomaterials (e.g., MXenes), covalent organic frameworks, and various charged polymers in solid-state or lithium/sodium-based nonflow systems.^13–15^ While these strategies demonstrate improved shuttle suppression, many rely on costly materials, elaborate synthesis steps, or environmentally hazardous solvents. Furthermore, most are designed for nonflow systems, and their effectiveness under realistic Na–S flow cell conditions remain limited.

Glass fiber (GF) membranes have been widely adopted in various battery systems including sodium–metal, lithium–metal, and zinc–air batteries due to their low cost, chemical stability, and mechanical flexibility. However, its inherently high porosity and electrochemically inert surface render it ineffective at restricting polysulfide crossover or controlling interfacial reactions.^16,17^ Pristine GF has been identified as a primary conduit for polysulfide crossover, leading to rapid cell degradation in previous studies.^18–23^ These limitations become even more pronounced under dynamic flow conditions, where continuous electrolyte convection through highly porous GF membranes accelerates polysulfide transport and interfacial degradation, rendering pristine GF separators unsuitable for Na–S NARFB applications. Although the intrinsic thickness of GF remains disadvantageous from a strict energy-density perspective, separator selection in Na–S NARFB must also consider polysulfide-shuttle suppression, chemical compatibility, mechanical robustness, processability, and cost. In this context, we view GF not as an optimal membrane in its pristine form, but as a low-cost and scalable scaffold that can be functionally upgraded through surface modification.

In this study, we designed a simple yet effective strategy to functionalize commercial GF separators for Na–S NARFBs applications without complex synthesis or fabrication pathways, using a water-based dip-coating process (Figure 1a). To the best of our knowledge, this work represents the first demonstration of GF membranes as viable separators for Na–S NARFBs, where their intrinsic high porosity has previously precluded practical use under flow conditions. As illustrated in the flow-cell schematic, the separator plays a central role in regulating polysulfide transport between the Na metal anode and the Na_2_S_8_ catholyte reservoir under dynamic flow conditions. To impart selectivity to this highly porous membrane, poly­(4-styrenesulfonic acid-*co*-maleic acid) sodium salt (PSSMA), a commercially available polyanionic polymer bearing sulfonate (−SO_3_^–^) and carboxylate (−COO^–^) groups, was selected as the surface-modifying agent (Figure 1b). PSSMA was selected because it combines dual anionic functionality with straightforward water-based processability, allowing a simple dip-coating route without the use of organic solvents. In addition, its commercial availability, PFAS-free composition, and practical compatibility with the fibrous GF scaffold make it an attractive polymer platform for scalable membrane functionalization. When coated onto GF, these anionic functionalities generate electrostatic repulsion toward negatively charged polysulfide species, while the conformal polymer coating partially densifies the fibrous network, reducing effective porosity and suppressing polysulfide crossover in the flow-cell configuration. In addition to regulating catholyte transport, the PSSMA-modified separator promotes a more uniform Na-ion flux at the sodium-metal interface, contributing to the formation of a stable PSSMA-derived SEI. The PSSMA–GF membrane was fabricated by immersing pristine GF into an aqueous PSSMA solution for 30 s, followed by drying at 50 °C (Figure 1c). The resulting membrane retained its macroscopic integrity and flexibility, exhibiting a uniform pale-yellow appearance without structural deformation. This facile, water-processable approach provides a scalable and environmentally benign route to impart ionic functionality and selective transport properties to GF separators for sodium polysulfide flow batteries.

**Figure 1:**
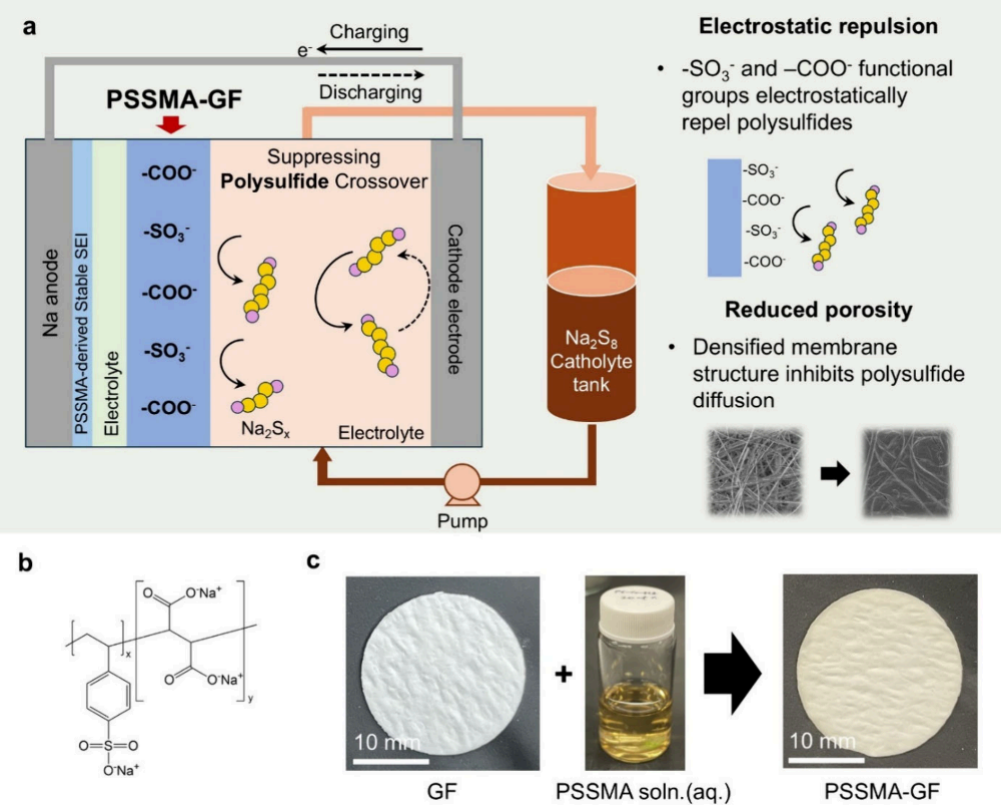
Design and fabrication of the PSSMA–GF membrane for shuttle suppression in sodium polysulfide flow batteries. (a) Schematic illustration of the Na|PSSMA–GF|Na_2_S_8_ flow cell. (b) Chemical structure of poly­(4-styrenesulfonic acid-*co*-maleic acid) sodium salt (PSSMA). (c) Photographs of pristine GF, PSSMA solution, and fabricated PSSMA–GF membrane.

## Results and Discussion

Considering the solubility range of PSSMA, we varied the concentration of the dip-coating solution from 5 to 40 wt % and analyzed the structural and physical properties of the resulting membranes. Scanning electron microscopy (SEM) images revealed that pristine GF exhibited a porous structure with large pores, whereas these pores were progressively filled as the PSSMA concentration increased (Figure 2a). At 40 wt %, most pores appeared densely packed. As the PSSMA concentration increased, the amount of polymer coated onto the GF also increased linearly (Figure 2c). To further quantify the structural change induced by the coating, the membrane thickness was measured for pristine GF and all PSSMA–GF membranes prepared with different PSSMA concentrations (Figure S1). Although the thickness increased only modestly with increasing PSSMA loading, the calculated apparent membrane density increased overall (Table S1). Representative cross-sectional SEM images (Figure S2) further support that the coating renders the fibrous membrane structure more compact.

**Figure 2:**
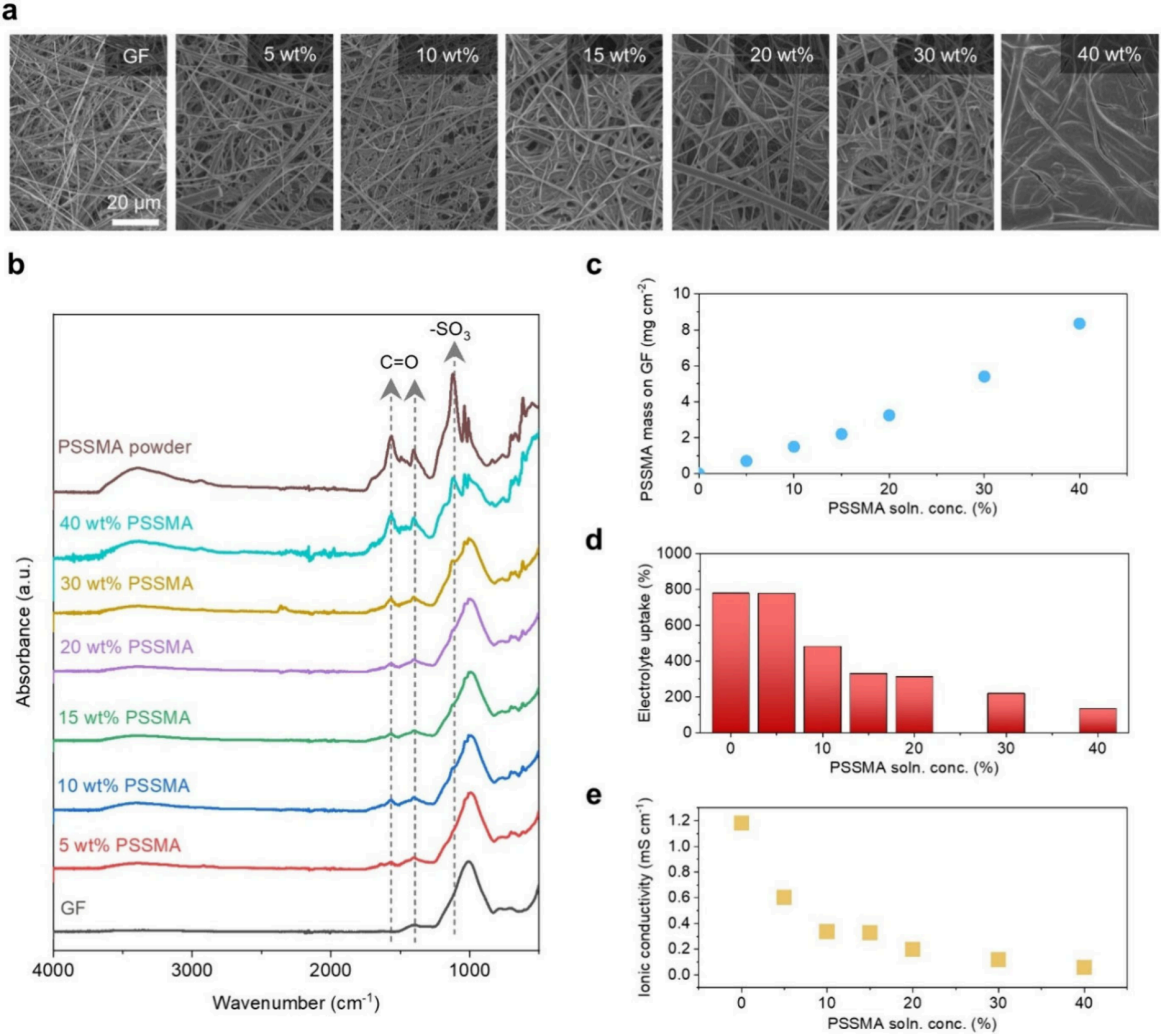
(a) SEM images of pristine GF and PSSMA–GF membranes fabricated with PSSMA solution concentrations of 5, 10, 15, 20, 30, and 40 wt %. All images are shown on the same scale. (b) FT-IR spectra of pristine GF, PSSMA powder, and PSSMA–GF membranes prepared with varying PSSMA concentrations. (c) Mass of PSSMA loaded onto GF, (d) electrolyte uptake of membranes, and (e) ionic conductivity of the membranes at room temperature as a function of the PSSMA solution concentration. For ionic conductivity measurements, 50 μL of liquid electrolyte was added to each membrane.

Fourier transform infrared spectroscopy (FT-IR) analysis confirmed this trend, with the absorbance intensities of characteristic peaks corresponding to −SO_3_^–^ (1119 cm^–1^) and −COO^–^ (1410 and 1567 cm^–1^) increasing proportionally with coating content (Figure 2b).^24^ Energy-dispersive spectroscopy (EDS) elemental mapping further confirmed that PSSMA was uniformly deposited over the entire GF surface, as evidenced by the even distribution of sulfur (S) and sodium (Na) signals (Figure S3). Electrolyte uptake of the membrane is closely related to its pore structure and functional group composition, which directly affects ion transport and redox species diffusion. As the PSSMA concentration increased, the porosity of the membrane decreased, leading to a gradual decrease in electrolyte uptake (Figure 2d). Nevertheless, the 40 wt % PSSMA–GF still maintained a high uptake of 134%, which is significantly higher than that of Nafion 212 (<50%), a representative PFAS-based ion-exchange membrane. This indicates that the balance between porosity and functional group incorporation was well achieved.^11^ The ionic conductivity of the membrane exhibited a gradual decline with increasing PSSMA content (Figure 2e). This was attributed to reduced porosity and smaller pore sizes limiting ion transport pathways, despite the presence of ion-conducting −SO_3_^–^ and −COO^–^ groups. Because the present membrane design relies on a water-soluble polyanionic coating, its retention under the nonaqueous tetraethylene glycol dimethyl ether (TEGDME) solvent is an important consideration. The PSSMA–GF membrane was therefore additionally characterized after soaking in the electrolyte solvent (Figure S4). Postsoaking SEM, EDS, and FT-IR analyses showed that the fibrous morphology and characteristic coating-related signals were largely preserved, with the S/Si ratio changing only modestly from 0.541 to 0.467, indicating substantial retention of the PSSMA coating after solvent exposure. Such retention is also reasonable from an interfacial materials perspective, because glass/silica-based surfaces are hydroxylated and polymer coating of GFs is a well-established surface-modification strategy.

The effectiveness of the PSSMA–GF membrane in suppressing polysulfide crossover was assessed by monitoring OCV retention and cycling stability in Na||0.25 M Na_2_S_8_ full cells. Based on subsequent performance comparisons shown in Figure S5, the PSSMA–GF membrane discussed herein refers to the sample prepared with a 30 wt % PSSMA solution, which exhibited the best overall performance. As shown in Figure 3a, full cells assembled with a pristine GF membrane exhibited a rapid drop in OCV within just a few minutes, indicative of severe polysulfide crossover. In contrast, cells with the PSSMA–GF membrane maintained nearly 100% of the initial OCV, confirming effective suppression of crossover. Remarkably, even membranes modified with only 5 wt % PSSMA showed comparable performance (Figure S5), highlighting that minimal surface functionalization is sufficient to achieve substantial improvement in crossover resistance.

**Figure 3:**
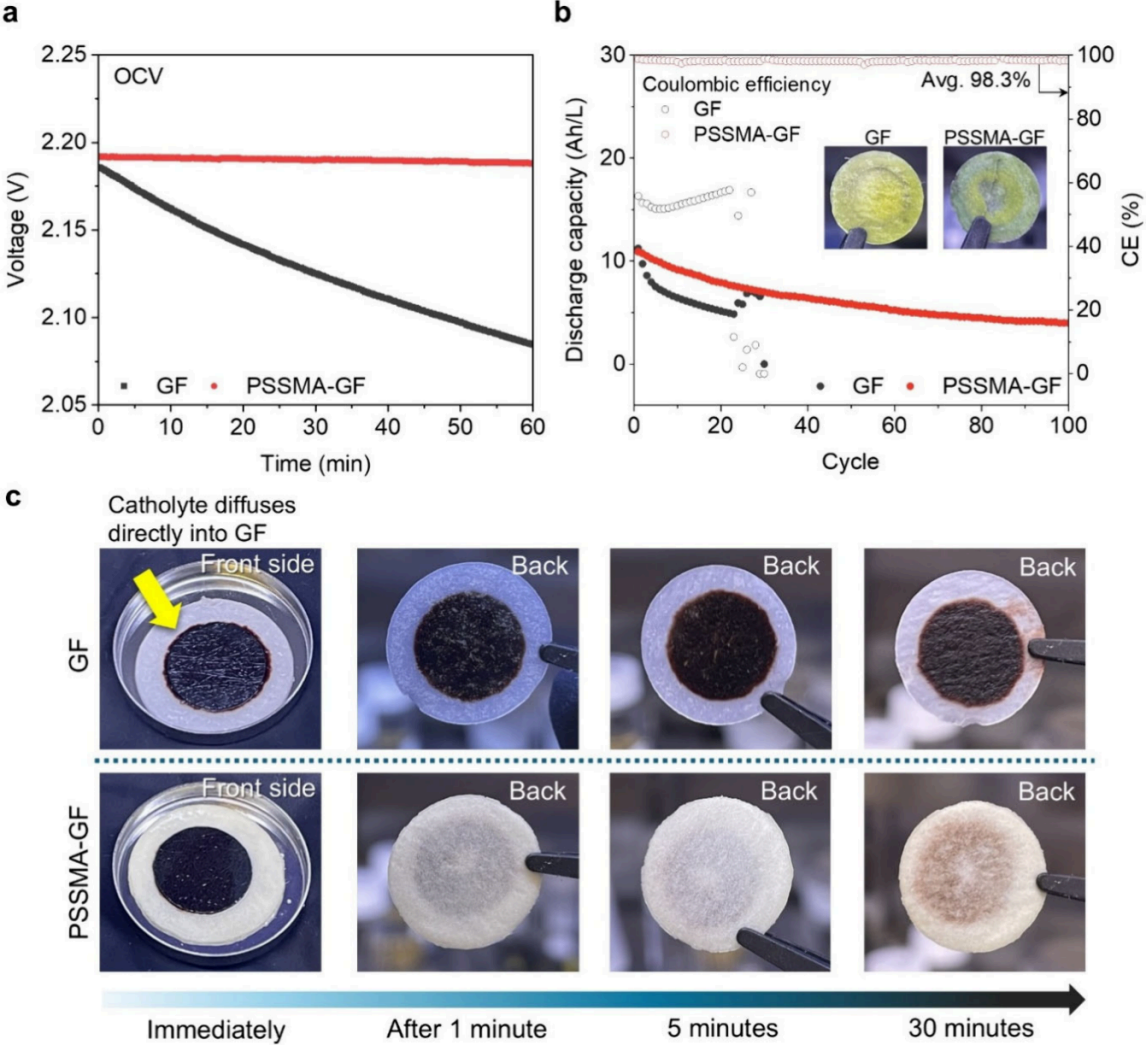
(a) OCV evolution of Na||Na_2_S_8_ full cells using GF and PSSMA–GF before cycling. (b) Long-term cycling performance at 0.33 mA cm^–2^. Insets show photographs of GF and PSSMA–GF (30%) membrane surfaces (facing the Na metal anode) after cycling. (c) Catholyte diffusion test. A carbon paper was placed on top of each membrane and catholyte was dropped onto it, mimicking a coin-cell assembly. Time-dependent catholyte diffusion was compared using photographs taken immediately after dropping, 1 min later, 5 min later, and 30 min later.

Furthermore, a diffusion test mimicking the coin-cell configuration was conducted to qualitatively assess the catholyte permeation behavior across different membranes (Figure 3c). In the case of the pristine GF, the polysulfide catholyte rapidly penetrated through the membrane and reached the backside within 1 min. This implies immediate contact with the sodium anode, which can lead to self-discharging and the formation of less soluble short-chain polysulfide on the sodium anode surface. In contrast, the PSSMA–GF membrane significantly delayed catholyte infiltration, with no complete permeation observed even after 30 min. This suggests that the introduction of electrostatic repulsion and reduced porosity effectively formed a physical and chemical repulsion barrier against polysulfide anions. These trends were consistent with electrochemical cell performance. Na||Na_2_S_8_ full cells using pristine GF exhibited steep capacity loss within the 2.2–2.3 V range during the initial cycles, and two out of three cells failed within just two cycles (Figure S6). This correlation underscores that rapid catholyte diffusion directly compromises cell stability, and highlights the critical role of membrane design in mitigating polysulfide shuttle effects and extending cycle life.

We further compared the long-term cycling performance of the most stable pristine GF-based and the PSSMA–GF Na–S full cells. At a current density of 0.33 mA cm^–2^, the PSSMA–GF-based cell exhibited significantly improved capacity retention and a high average CE of 98.3% (Figure 3b). The inset of Figure 3b shows the membrane surfaces facing the sodium metal after cycling: the GF membrane appears entirely yellow due to the precipitation of short-chain polysulfides, while the PSSMA–GF membrane retains a greenish color with only partial yellowing, indicating effective suppression of polysulfide crossover. While the GF-based cell showed comparable initial discharge capacity, it exhibited a rapid capacity fade in subsequent cycles due to significant polysulfide crossover during the charging process, resulting in a low CE (<60%). The performance degradation of the GF cell likely stems from the initial loss of active materials due to polysulfide crossover and continuous shuttling behavior. These findings underscore the limitations of pristine GF membranes in flow battery systems that require high polysulfide concentrations and highlight the practical value of our surface modification strategy. During the first cycle, the PSSMA–GF-based cell exhibited a noticeable overpotential upon charging, which related to the reduced porosity of the coated membrane and the need for an activation process (Figure S7a).

To further examine the membrane after prolonged cell operation, post-mortem characterization was carried out on the PSSMA–GF separator recovered after 100 cycles of the Na||Na_2_S_8_ full cell (Figure S8). The recovered membrane retained its fibrous framework in the SEM image, while postcycling FT-IR still showed the characteristic C=O- and −SO_3_-related features of PSSMA, indicating that the polymer-derived chemical functionality remained detectable after long-term cycling. EDS mapping further confirmed the continued presence of O, C, S, and Na signals across the membrane.

Electrochemical impedance spectroscopy (EIS) of the PSSMA–GF cell revealed a slightly increased in the initial solution resistance due to the membrane coating, but the total resistance remained stable after cycling (Figure S9). In contrast, the GF-based cell showed unstable impedance behavior due to rapid cell failure induced by polysulfide shuttling. When membranes with different PSSMA concentrations were evaluated, the 30 wt % PSSMA–GF membrane showed the best long-term cycling performance of full cells (Figure S10). Lower concentrations resulted in insufficient blocking of polysulfides, whereas higher concentrations compromised ion transport due to excessive pore blockage. To further extend the electrochemical evaluation beyond the single-current-density condition, additional tests were performed using the PSSMA–GF-based Na||Na_2_S_8_ full cell at multiple current densities (Figure S11). The cell delivered measurable capacities over the tested current-density range in the rate-performance test, and long-term cycling was further examined at 0.22, 0.66, and 0.99 mA cm^–2^. These results broaden the electrochemical evaluation of the PSSMA–GF membrane under different operating conditions. Because the PSSMA–GF membrane exhibits lower electrolyte uptake than pristine GF, we further examined whether the full-cell performance could be maintained under reduced electrolyte conditions, which are more relevant to practical cell operation. As shown in Figure S12, 10 μL of electrolyte was insufficient to fully wet the membrane, whereas 20 μL or more provided complete wetting. Correspondingly, the delivered capacity increased with electrolyte volume, indicating that the PSSMA–GF membrane can operate under reduced electrolyte loading, provided that sufficient wetting is maintained in the present cell configuration.

Cyclic voltammetry (CV) analysis of the Na|PSSMA–GF|Na_2_S_8_ cell revealed three cathodic and three anodic peaks, indicating the reversible redox transitions of various polysulfide species (Figure S7b). In contrast, the GF-based cell could not generate a valid CV curve due to severe cell degradation caused by excessive initial crossover. In addition, differential capacity (d*Q*/d*V*) analysis showed a shift in the reduction peak of the PSSMA–GF-based cell toward lower potentials, which is consistent with delayed conversion of intermediate polysulfides into lower-order insoluble species under membrane-regulated transport conditions (Figure S13).

In Na|Na symmetric cell tests, the pristine GF-based cell exhibited a slightly lower initial overpotential compared to the PSSMA–GF cell, which can be attributed to the higher porosity and lower initial ionic resistance of the unmodified GF separator. However, this behavior was not sustained. The GF-based symmetric cell experienced an abrupt short circuit within approximately 12 h of cycling, indicating rapid interfacial degradation and unstable sodium deposition. In contrast, the PSSMA–GF-based cell maintained stable voltage profiles over prolonged cycling without short-circuit failure (Figure S14). The early failure of the GF-based cell suggests that the absence of interfacial regulation leads to nonuniform Na-ion flux and localized current concentration, which promotes uncontrolled sodium growth rather than the formation of a stable SEI. This interpretation is supported by X-ray photoelectron spectroscopy (XPS) analysis of the sodium surface after cycling.

As shown in Figure S15, the sodium electrode cycled with the pristine GF separator exhibits a pronounced Cl signal, indicative of extensive NaClO_4_ decomposition and the formation of a chemically heterogeneous, inorganic-rich interphase. By contrast, the PSSMA–GF cell shows suppressed Cl-related species together with enhanced C–O-based components in the SEI, indicating moderated electrolyte decomposition and the active involvement of PSSMA-derived carboxylate groups in interphase formation. This observation suggests that the carboxylate functionalities undergo reductive transformation below ∼1 V, contributing to the development of a thin and chemically homogeneous interphase (Figure S16). These organic species are consistent with the formation of a thin and chemically uniform SEI layer that passivates the sodium surface while maintaining ion transport. The coexistence of PSSMA-derived organic components with reduced inorganic salt decomposition suggests that the PSSMA–GF separator does not merely block polysulfide transport but also contributes directly to chemically regulated SEI formation. Taken together, these results demonstrate that a lower initial overpotential does not necessarily correlate with interfacial stability in sodium-metal systems. Instead, sustained cycling stability is governed by controlled ion transport and chemically regulated SEI chemistry, both of which are enabled by the PSSMA-coated separator.

The polysulfide shuttling suppression capability of the PSSMA–GF membrane was quantitatively evaluated by measuring the shuttle current and self-discharge behavior.^25,26^ The shuttle current originates from the repeated diffusion and reduction of soluble polysulfide species at the counter electrode during cycling, acting as a parasitic current and representing a major source of performance degradation in sulfur batteries.^27^ Higher shuttle currents typically lead to reduced CE, electrolyte consumption, and parasitic reactions at the anode, thereby shortening the cycle life. To assess this, Na||Na_2_S_8_ cells were cycled three times, followed by a stepwise voltage sweep from 1.9 to 2.3 V to measure the resulting shuttle current (Figure 4a). In the case of the GF membrane, the shuttle current increased sharply with increasing voltage, indicating accelerated polysulfide crossover and reduction. In contrast, the PSSMA–GF cell maintained near-zero shuttle current across the entire voltage range, suggesting that the electrostatic repulsion provided by the sulfonate (−SO_3_^–^) and carboxylate (−COO^–^) groups, along with reduced porosity, effectively inhibited polysulfide transport. To further examine the membrane’s suppression of self-discharge, we monitored the OCV after resting the fully charged full cell for a given period (Figure 4b). Self-discharge primarily arises from the migration of long-chain polysulfides to the anode, where they are reduced into lower-order and less soluble species, leading to a drop in cell voltage. The GF-based cell exhibited a rapid decline in OCV within a short period, while the PSSMA–GF cell maintained a stable charged voltage, reaffirming its ability to mitigate polysulfide shuttling. The Na^+^ transference number increased from 0.33 for pristine GF to 0.65 for PSSMA–GF (Figure S17), indicating more sodium-ion-favored transport after PSSMA coating. This trend is consistent with the fixed anionic groups in PSSMA, which make anionic transport less favorable while still allowing Na^+^ conduction through the electrolyte-filled membrane.

**Figure 4:**
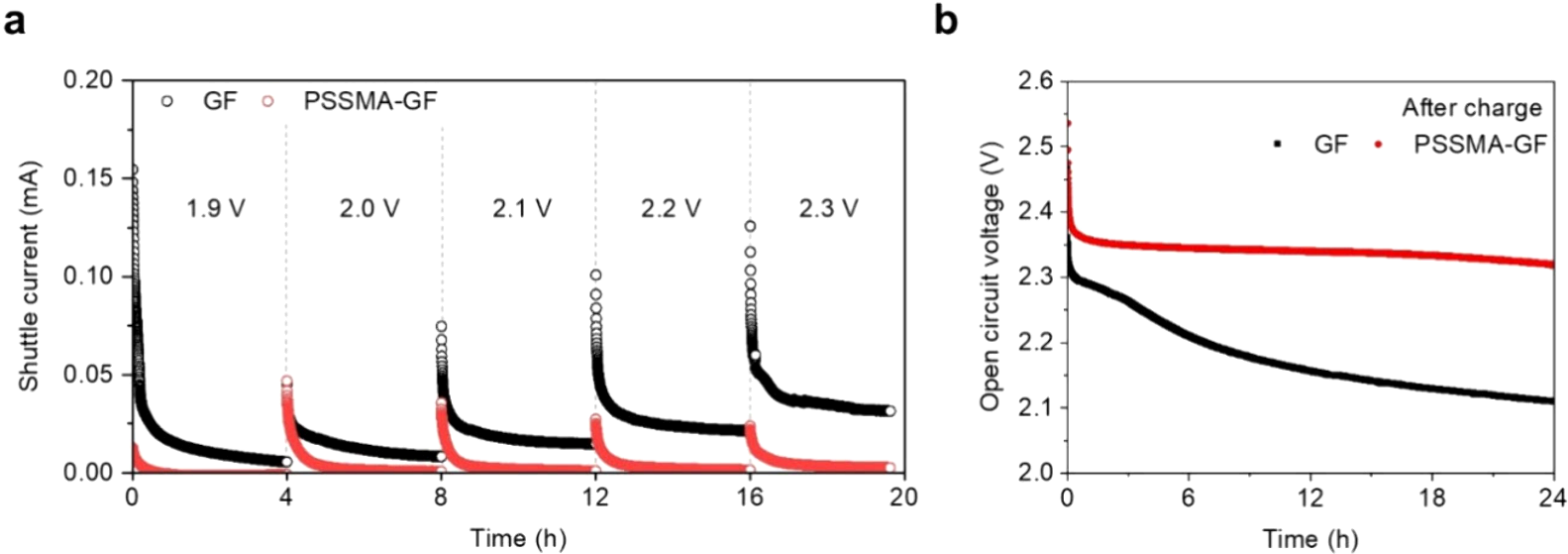
(a) Shuttle current profiles of Na||Na_2_S_8_ full cells with GF and PSSMA–GF under different potentiostatic voltages after three cycles. (b) OCV retention after charging to evaluate self-discharge behavior.

To further compare the PFAS-free PSSMA coating with a fluorine-rich control, a poly­(vinylidene fluoride)-coated glass-fiber membrane (PVDF-GF) with a comparable polymer loading was additionally prepared and evaluated (Figure S18). Although PVDF-GF showed partial improvement over pristine GF in shuttle current, OCV retention, and long-term cycling, its overall performance remained inferior to that of PSSMA–GF. This comparison suggests that the advantage of PSSMA–GF is not simply due to the presence of a polymer coating, but is more closely related to the specific chemistry of the fixed polyanionic layer.

To further distinguish fixed-charge-mediated selectivity from simple structural blocking, additional polymer-coated control membranes, including poly­(vinyl alcohol)-coated GF (PVA-GF) and polystyrene-*block*-polyisoprene-*block*-polystyrene-coated GF (PSIS-GF), were prepared with polymer loadings comparable to that of PSSMA–GF and compared with pristine GF and PSSMA–GF (Figure S19). PVA was selected as a nonionic hydrophilic polymer that can be processed through a water-based dip-coating route similar to that used for PSSMA, allowing us to examine whether the improvement could arise simply from coating a water-processable polymer onto GF. PSIS was selected as a useful styrenic but nonionic control, allowing the role of the fixed anionic groups in PSSMA to be distinguished from that of a styrenic polymer coating alone. Although all three polymer-coated controls provided partial improvement over pristine GF, none matched the shuttle suppression or long-term cycling performance of PSSMA–GF, indicating that the advantage of PSSMA–GF cannot be explained by geometric blocking alone and is instead associated with the combined effects of structural densification and fixed-charge-mediated transport regulation.

In addition, to validate the versatility of our approach, we applied the same dip-coating procedure to a commercial PVDF membrane (Figure S20). The resulting Na||Na_2_S_8_ full cell exhibited improved OCV retention and enhanced cycling performance, demonstrating that the PSSMA coating strategy can overcome limitations of conventional membranes and is broadly applicable to various porous fibrous substrates.

Finally, to demonstrate the practical applicability of the PSSMA–GF membrane beyond coin-cell configurations, a Na–S NARFB was demonstrated in Na|PSSMA–GF|0.25 M Na_2_S_8_ configuration (Figure 5a). Figure S21 shows the EIS spectrum of the flow cell measured prior to cycling. The relatively low real-axis intercept in the high-frequency region indicates a small bulk and contact resistance under flowing electrolyte conditions, which is mainly attributed to continuous electrolyte circulation and flow-assisted ion/mass transport in the flow-cell configuration. The RFB was operated at a current density of 1 mA cm^–2^, which is approximately three times higher than that used in the coin-cell tests, to evaluate membrane performance under more demanding conditions. As shown in Figure 5b, the first discharge profile exhibits a clear and stable voltage plateau at around 1.5 V, consistent with that observed in coin-cell measurements, indicating that the PSSMA–GF membrane preserves the fundamental polysulfide redox behavior under flow conditions. The cell exhibited an OCV of 2.04 V and delivered a first-discharge capacity of 4.12 Ah L^–1^, calculated based on the catholyte volume. Based on the catholyte volume, this corresponds to an energy density of approximately 8.4 Wh L^–1^ (Table S2). Because the present cell employs a Na metal anode in a hybrid flow-battery configuration, rather than a conventional anolyte tank, this architecture is inherently attractive from an energy-density perspective, although the present value should be understood as a catholyte-based metric rather than a full system-level energy density.

**Figure 5:**
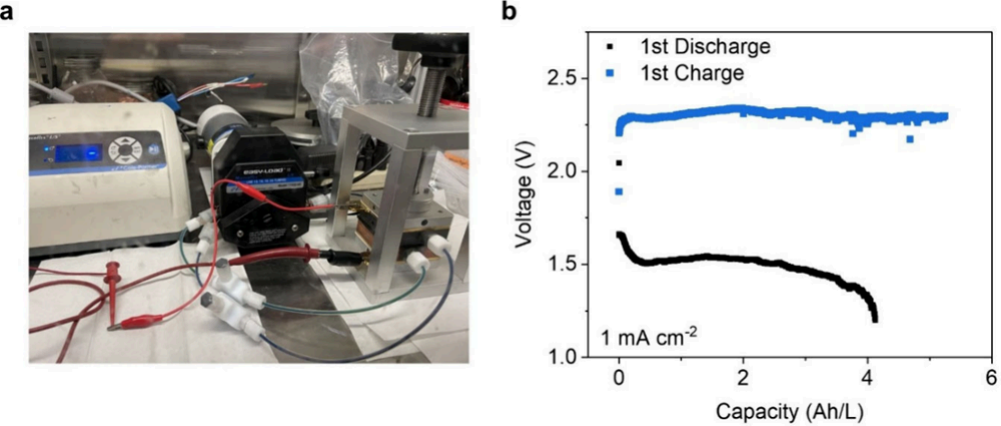
(a) Photograph of the Na|PSSMA–GF|Na_2_S_8_ flow cell during electrochemical operation. (b) Galvanostatic voltage profiles of the first discharge and first charge of the Na|PSSMA–GF|Na_2_S_8_ flow cell.

During the subsequent charge process, the voltage plateau increased and stayed at 2.27 V as expected, which indicates a 0.75 V overpotential under 1.0 mA cm^–2^ using flow-cell configuration. Despite remaining engineering-related challenges associated with flow-cell configurations, the successful first-discharge behavior demonstrates the feasibility of employing the PSSMA–GF membrane in practical flow-cell systems. This preliminary result suggests that electrostatic regulation via PSSMA coating can be extended to flow environments at the proof-of-feasibility level. At the same time, because the present membrane is based on a relatively thick fibrous scaffold, improved shuttle suppression is accompanied by some transport penalty, as reflected by the reduced ionic conductivity, lower electrolyte uptake, and increased initial overpotential after coating. In this regard, the optimized PSSMA–GF membrane should be understood as a balance between selectivity and ion transport rather than as an application-optimized membrane architecture, and future benchmarking against more established membrane platforms used in the same sodium polysulfide flow-battery chemistry will be necessary.

## Conclusions

In this study, we present a simple and effective membrane modification strategy that enables the practical use of GF separators in Na–S NARFBs, addressing the key challenges of polysulfide shuttling and sodium–metal interfacial instability. By dip-coating a commercial GF membrane with a water-soluble PSSMA, ionic functionality and controlled physical blocking are introduced while preserving the mechanical advantages of the GF scaffold. The optimized PSSMA–GF membrane maintains high electrolyte uptake and sufficient ionic conductivity, and effectively suppresses polysulfide crossover, leading to enhanced cycling stability, high CE, and improved interfacial robustness in full-cell tests. These advantages are quantitatively validated through shuttle current measurements, self-discharge analysis, and differential capacity evaluation. To further assess practical applicability, a preliminary sodium-polysulfide flow-cell demonstration was conducted using the PSSMA–GF membrane. The flow cell exhibits a stable discharge plateau and representative capacity under elevated current density, indicating that the electrostatic regulation imparted by the PSSMA coating remains effective under dynamic flow conditions. Although flow-cell engineering limitations still need to be addressed, this result confirms the feasibility of extending the membrane design beyond coin-cell configurations. Overall, this PFAS-free, water-processable, and scalable dip-coating strategy offers a practical pathway for advancing membrane technologies in high-energy-density sodium-polysulfide flow battery systems.

## Materials and Methods

### Materials

Poly­(4-styrenesulfonic acid-*co*-maleic acid) sodium salt (PSSMA, average *M*_w_ ≈ 20000), sodium metal, sodium sulfide (Na_2_S, 98%), sulfur powder (S, 99.98% trace metals basis), and tetraethylene glycol dimethyl ether (TEGDME, ≥99%) were purchased from Sigma-Aldrich. Sodium perchlorate (NaClO_4_, 98.0–102.0%) was purchased from Fisher Scientific. Carbon paper (AvCarb P50, 0.007″ thickness) was purchased from Graphite Store. All the materials were used without further purification. Sodium polysulfide (Na_2_S_8_) was synthesized following the previous report.

### Fabrication of PSSMA–GF Membrane

Glass fiber (GF) membranes (Whatman GF6) were cut into 16-mm-diameter disks and dried under vacuum at 50 °C overnight before coating. PSSMA was dissolved in deionized water at various concentrations (5–40 wt %) and stirred for 12 h to ensure complete dissolution. The GF membranes were dip-coated in PSSMA solution for 30 s, followed by drying at 50 °C in a vacuum oven.

### Materials Characterization

The PSSMA loading on the membranes was evaluated by mass difference before and after coating. Surface morphologies were examined by field-emission scanning electron microscopy (SEM, Hitachi SU8230). Fourier transform infrared (FT-IR) spectroscopy (Nicolet 6700) was performed to confirm the presence of sulfonate (−SO_3_^–^) and carboxylate (−COO^–^) functional groups. Electrolyte uptake was measured by immersing the membranes in 1 M NaClO_4_ in TEGDME for 24 h and then calculating the weight increase relative to dry mass. X-ray photoelectron spectroscopy (XPS; Thermo Scientific K-Alpha) was used to analyze the SEI composition on sodium surfaces after cycling.

### Electrochemical Measurement

All cell assembly and measurements were conducted in an argon-filled glovebox. Ionic conductivity was determined by sandwiching the membranes between two stainless steel electrodes in a CR2032 coin cell and measuring impedance with a Biologic VMP3 potentiostat from 1 MHz to 1 Hz using a 10 mV AC signal at 25 °C. Na|Na symmetric cells and Na||Na_2_S_8_ full cells were assembled using CR2032 coin cells. For full cells, 10 mm carbon paper loaded with 12 μL of 0.25 M Na_2_S_8_ in 1 M NaClO_4_ in TEGDME was used as the cathode. The anode was a 12 mm sodium-metal disk, and the membrane was placed in between. For symmetric cells, two sodium-metal electrodes were used with 50 μL of electrolyte. Galvanostatic cycling tests were conducted at 0.33 mA cm^–2^ with voltage cutoffs from 1.2 to 2.8 V. CV was performed at 0.1 mV s^–1^. Shuttle current measurements were carried out after three cycles by applying potentiostatic voltages between 1.9 and 2.3 V and recording the steady-state current. Self-discharge tests were performed by charging the full cells to 2.8 V, then monitoring the OCV over time. Linear-sweep voltammetry (LSV) was performed using a stainless steel|PSSMA–GF|sodium configuration to assess electrochemical stability.

### Flow Cell Test

A Na–S RFB was assembled to evaluate the Na|PSSMA–GF|0.25 M Na_2_S_8_ configuration. This Na–S NARFB consisted of a copper anode chamber and a machined graphite cathode chamber. Both electrodes are crafted with a 2 × 2 cm^2^ serpentine flow channel in the center. During the cell assembling, a piece of 4 cm^2^ square sodium metal was made by hand-rolling to roughly 2 mm. A piece of copper sheet was placed between the copper anode chamber and the sodium metal to prevent metal deformation and blockage of the flow channels. At the graphite cathode side, two pieces of 4 cm^2^ square graphite felts (AvCarb G280A) were stacked and installed as the cathode current collector. A piece of PSSMA–GF membrane was cut into roughly 3 × 3 cm^2^ and used as the separator between the two electrodes. All flow-cell components were vacuum-dried overnight at 50 °C before moving into the glovebox for assembly. The anolyte reservoir contained 7 mL of 1.0 M NaClO_4_ in TEGDME, and the catholyte reservoir contained 6 mL of 0.25 M Na_2_S_8_ with 1.0 M NaClO_4_ in TEGDME. The electrolyte was circulated using a peristaltic pump at a revolution per minute at 6 (around 5–6 mL min^–1^). Galvanostatic cycling was performed with a Biologic potentiostat under a current density of 1 mA cm^–2^ within a voltage window of 1.2–2.8 V. The precycle EIS spectra were collected under the frequency range from 1 MHz to 200 mHz.

## Supplementary Material



## Notes

Membrane thickness and apparent density, cross-sectional SEM images, EDS mapping of PSSMA–GF, stability of a PSSMA coating after TEGDME soaking, OCV retention of PSSMA–GF membranes with different PSSMA loadings, electrochemical performance of pristine GF-based cells, charge–discharge profiles and cyclic voltammograms of PSSMA–GF-based cells, postmortem characterization of cycled PSSMA–GF membranes, EIS analysis, rate and long-term cycling performance at different current densities, reduced-electrolyte tests, differential capacity analysis, Na|Na symmetric cell tests, XPS analysis of SEI layers, LSV profiles, sodium-ion transference number measurements, fluorine-rich and neutral polymer control membranes, PSSMA coating on PVDF membranes, flow-cell EIS, and comparison of reported sodium polysulfide hybrid redox-flow battery systems (PDF)
