# Supplementary material for: Suppressing Polysulfide Crossover in Sodium Polysulfide Redox-Flow Batteries with an Oxyanion-Functionalized Glass Fiber Separator
Source: ACS Nano. 2026 Jun 27;20(27):19401–9. doi: 10.1021/acsnano.6c04702 (PMC13374493; doi:10.1021/acsnano.6c04702)
Supplement: Supplementary file 1 [file nn6c04702_si_001.pdf]

Supporting Information

Suppressing Polysulfide Crossover in Sodium-Polysulfide Redox Flow Batteries with an Oxyanion-functionalized Glass Fiber Separator

Jieun Kang,[a] Cheng-Tien Hsieh,[a] Wenda Wu,[b] Guang Yang,[b,*] and Nian Liu [a,*]

[a] School of Chemical and Biomolecular Engineering, Georgia Institute of Technology, Atlanta, Georgia 30332, United States

[b] Chemical Sciences Division, Oak Ridge National Laboratory, Oak Ridge, Tennessee 37831, United States

Corresponding Author

*E-mail. yangg@ornl.gov (Dr. Yang)

*E-mail. nian.liu@chbe.gatech.edu (Dr. Liu)

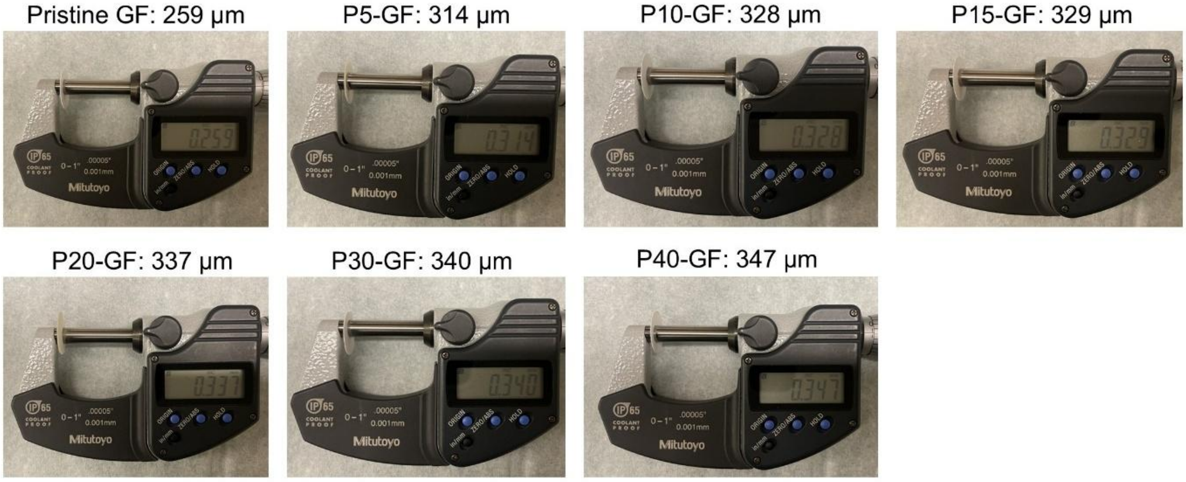

Figure S1. Thicknesses of pristine GF and PSSMA-GF membranes prepared using different PSSMA solution concentrations, measured with a thickness gauge. In P*n*-GF, "*n*" denotes the PSSMA solution concentration (wt%).

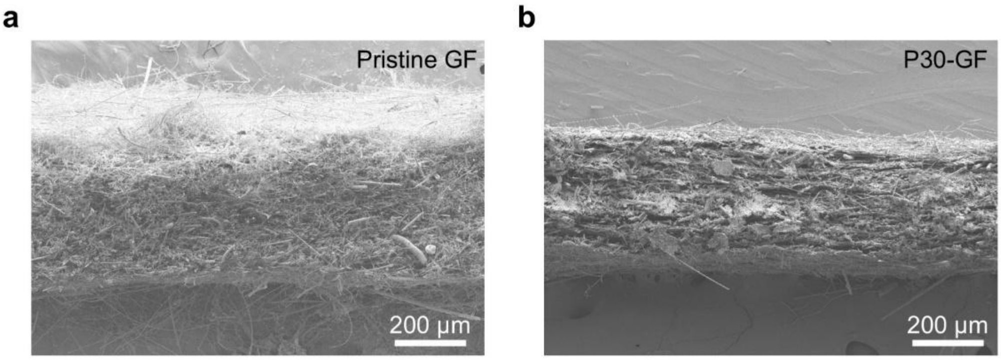

Figure S2. Representative cross-sectional SEM images of (a) pristine GF and (b) P30-GF. The pristine GF membrane exhibits a loose and highly porous fibrous network, whereas the PSSMA-coated membrane shows a more compact structure with partially reduced inter-fiber void space.

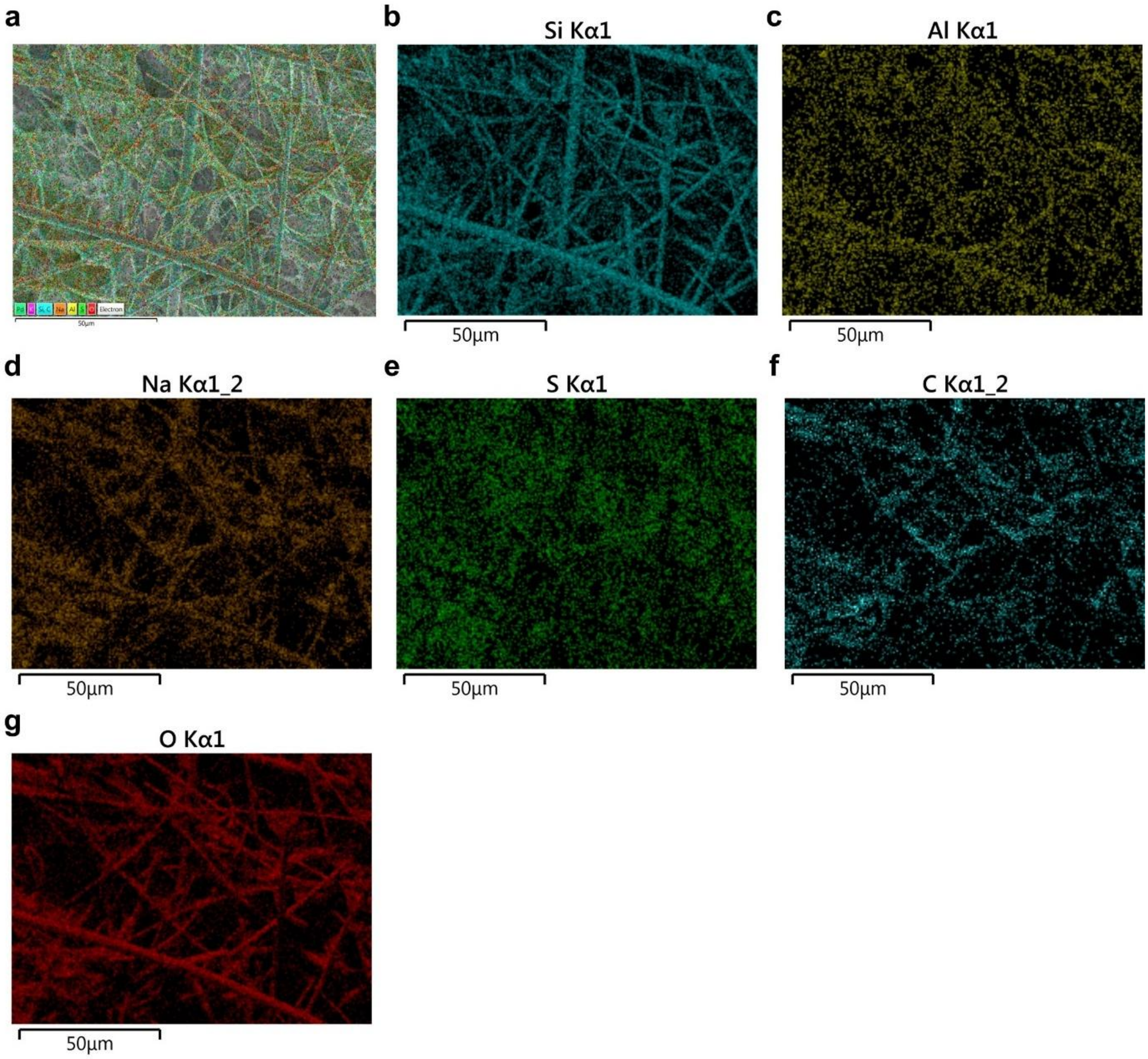

Figure S3. EDS mapping images of PSSMA-GF prepared using a 30 wt% PSSMA solution. (a) Overlay of all elements. Elemental mappings of (b) Si, (c) Al, (d) Na, (e) S, (f) C, and (g) O. The uniform distribution of Na, S, and C across the glass fiber confirms homogeneous PSSMA coating on the GF membrane.

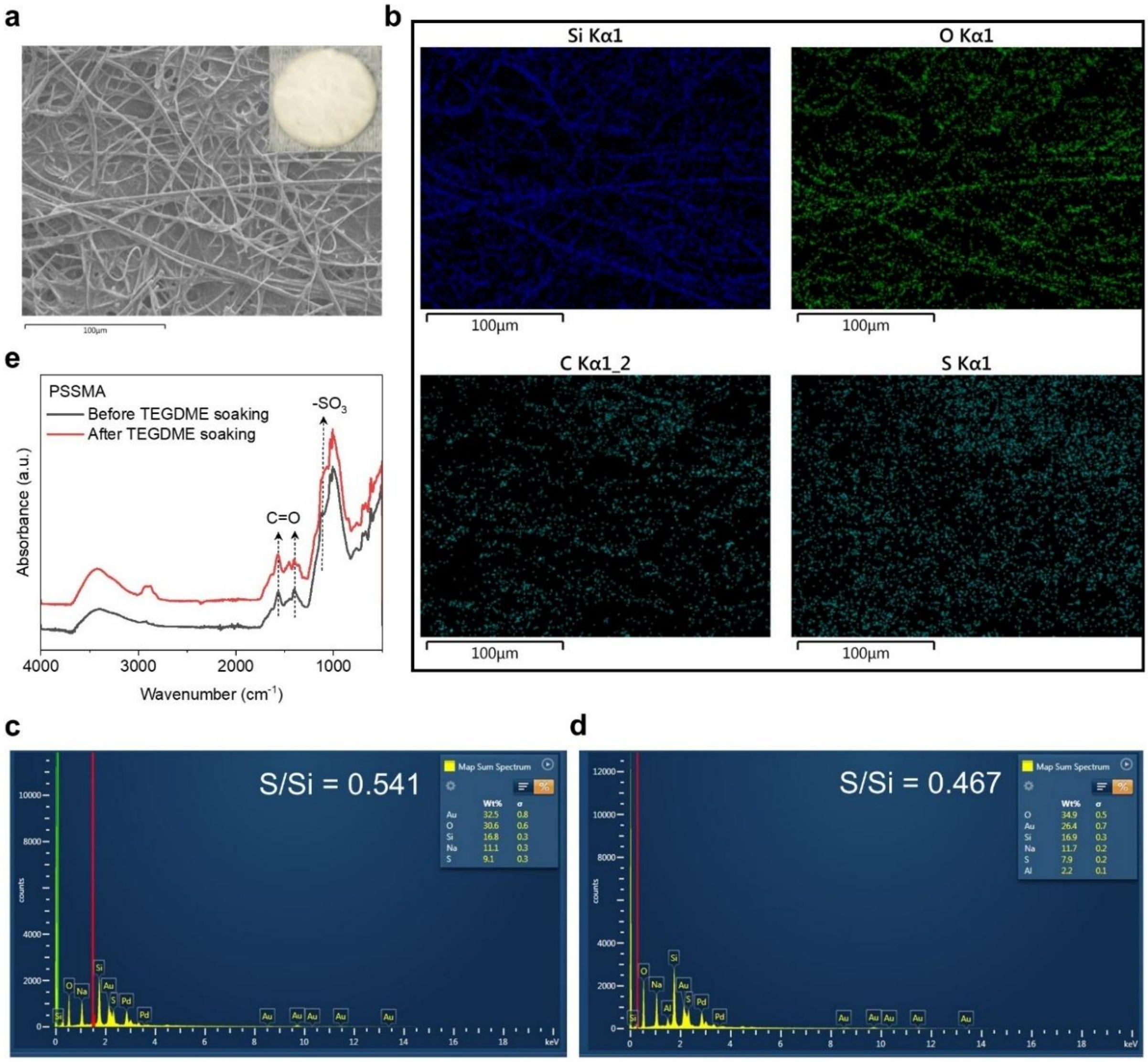

Figure S4. Stability of the PSSMA coating on GF after TEGDME soaking. (a) SEM image of the PSSMA-GF membrane after soaking in the TEGDME-based electrolyte and drying, with a photograph of the membrane shown in the inset. (b) Corresponding elemental mapping images of Si, O, C, and S, showing retention of the sulfur-containing coating on the glass-fiber framework. (c, d) Map-sum EDS spectra of the PSSMA-GF membrane before and after TEGDME soaking, respectively. The S/Si ratio remains in a comparable range after soaking. (e) FT-IR spectra of the PSSMA-GF membrane before and after TEGDME soaking, showing the persistence of characteristic sulfonate- and carboxylate-related bands.

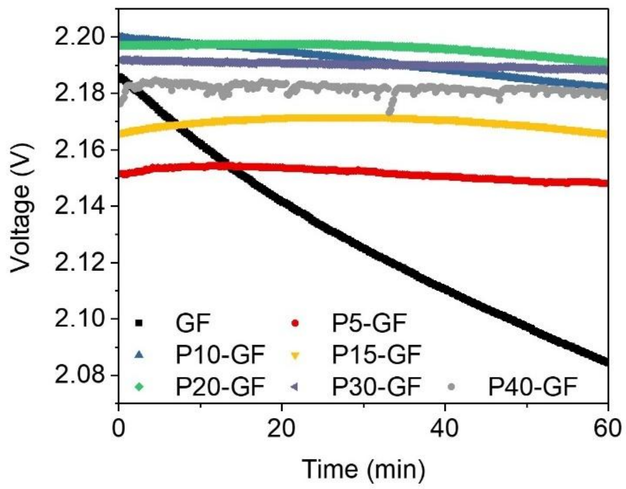

Figure S5. OCV changes of Na-Na_2S_8 full cells using PSSMA-GF membranes prepared with different PSSMA solution concentrations. In P*n*-GF, "*n*" denotes the PSSMA solution concentration (wt%). A pronounced improvement in OCV retention is observed even at low PSSMA loadings.

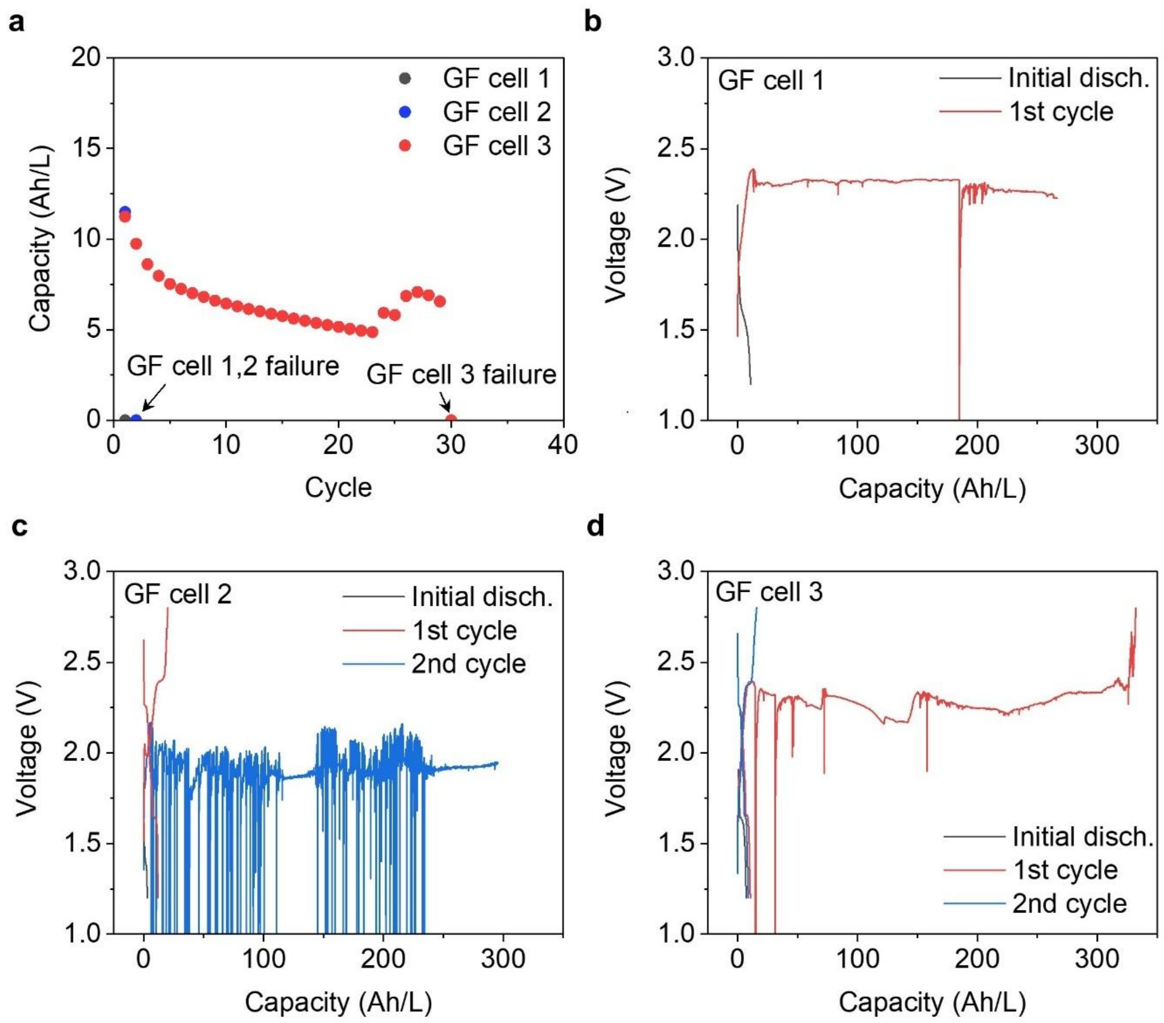

Figure S6. Electrochemical performance of three Na||Na_2S_8 full cells using pristine GF membranes. (a) Long-term cycling performance. (b-d) Initial charge–discharge voltage profiles of each individual cell. Two out of three GF-based full cells failed within two cycles, highlighting the severe instability of pristine GF membranes under polysulfide-rich conditions.

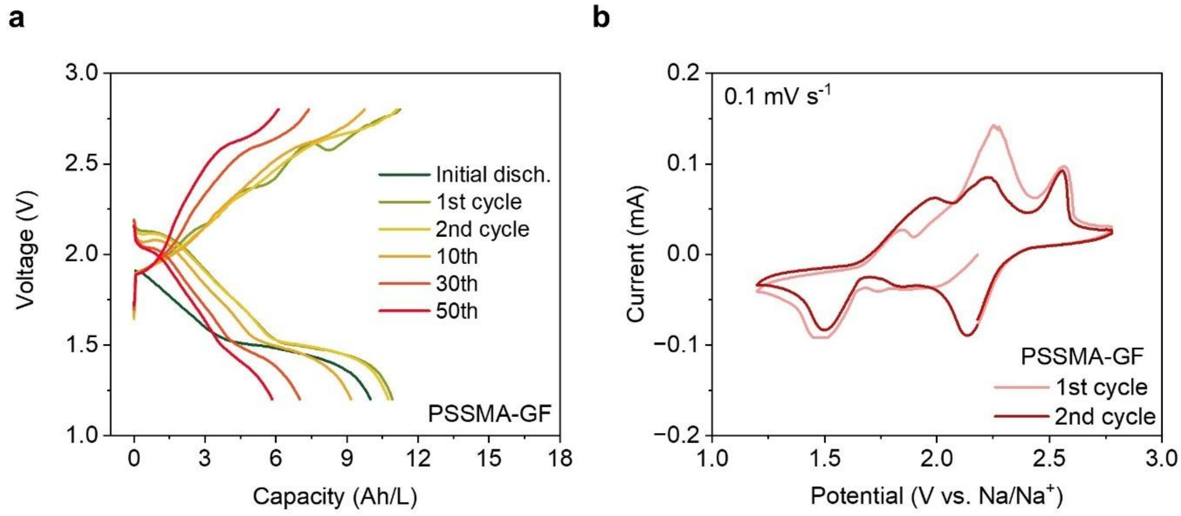

Figure S7. (a) Corresponding galvanostatic charge-discharge voltage profiles of Na||Na_2S_8 cell with PSSMA-GF membrane. (b) Cyclic voltammograms of Na||Na_2S_8 full cell with PSSMA-GF at a scan rate of 0.1 mV s^{-1}. The voltage profiles and multiple redox peaks confirm reversible polysulfide redox reactions enabled by the PSSMA-GF membrane.

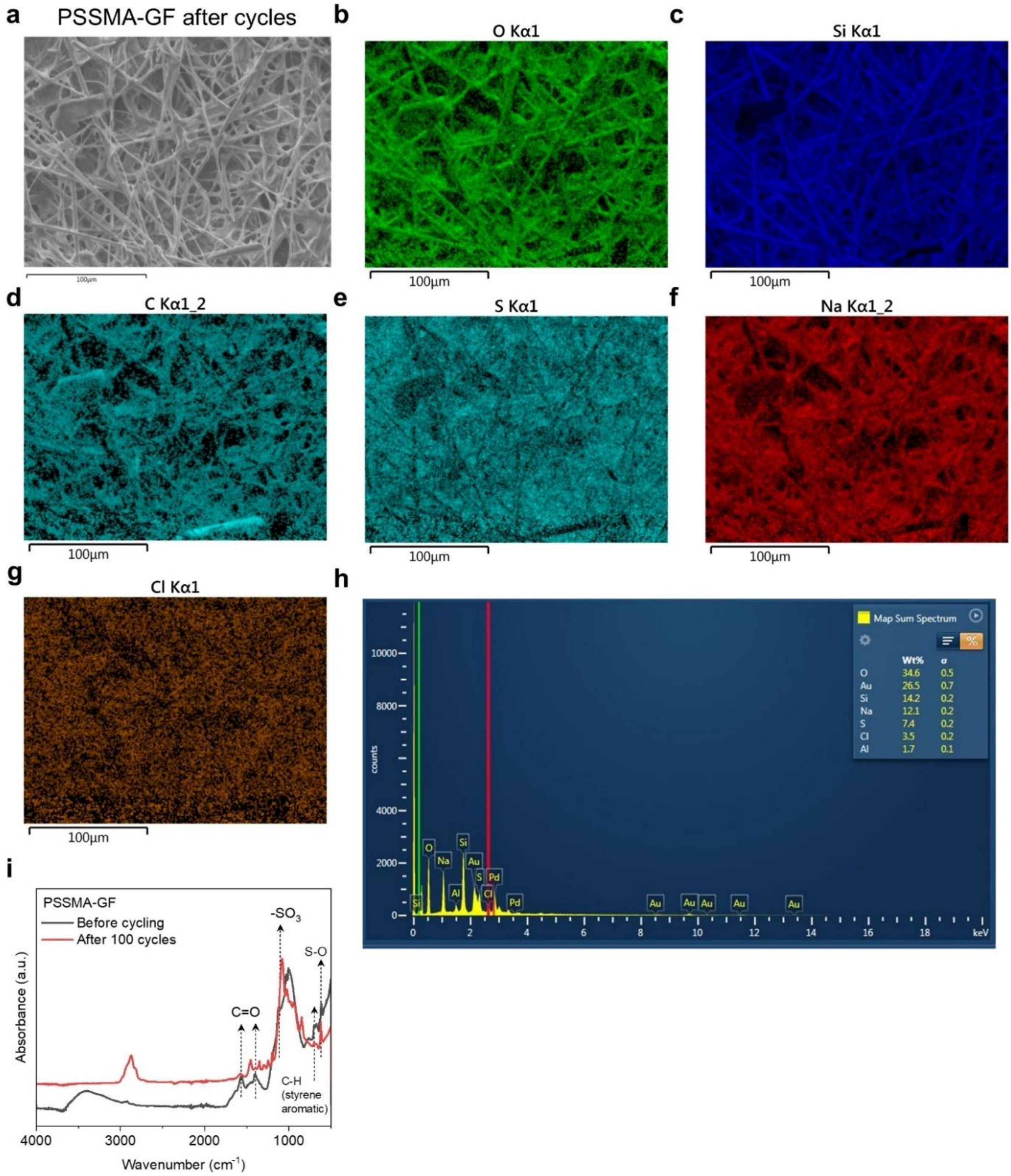

Figure S8. Post-mortem characterization of the PSSMA-GF membrane recovered after 100 cycles of the Na||Na_2S_8 full cell. (a) SEM image of the cycled membrane. (b-g) EDS elemental mapping images of O, Si, C, S, Na, and Cl. (h) Corresponding map-sum EDS spectrum of the cycled membrane. (i) FT-IR spectra of the PSSMA-GF membrane before cycling and after 100 cycles. The cycled membrane retains the characteristic C=O- and -SO_3-related features of PSSMA, together with low-wavenumber bands associated with the sulfonate-containing styrenic framework, although additional contributions from cell-derived species are also present after cycling.

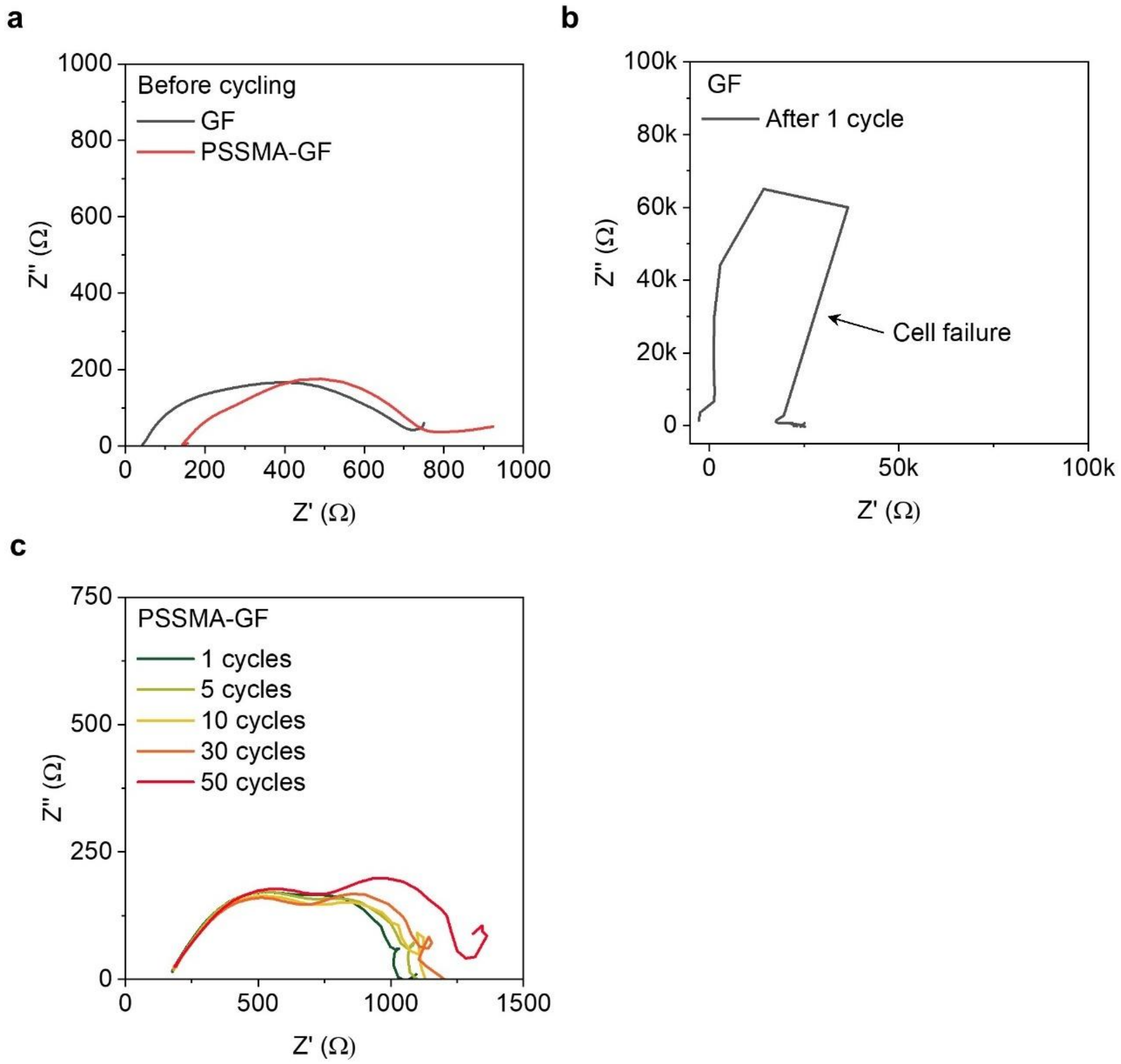

Figure S9. (a) Electrochemical impedance spectroscopy (EIS) spectra of Na||Na_2S_8 full cells with GF and PSSMA-GF membranes before cycling. (b) EIS spectrum of the GF-based cell after the first cycle. (c) EIS spectra of the PSSMA-GF-based full cell at different cycling stages. The PSSMA-GF cell shows a slightly increased initial solution resistance due to the membrane coating but maintains stable total impedance upon cycling, whereas the GF-based cell exhibits unstable impedance evolution associated with rapid failure caused by polysulfide shuttling.

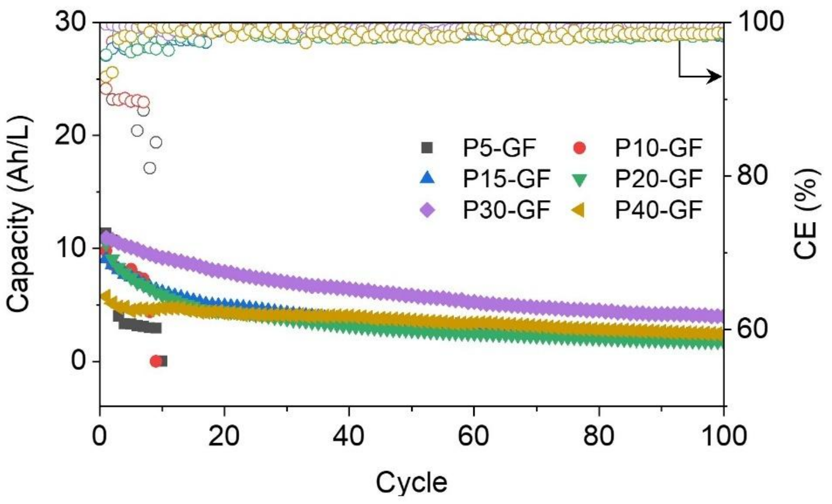

Figure S10. Long-term cycling performance of Na||Na_2S_8 full cells with PSSMA-GF membranes of varying PSSMA content at a current density of 0.33 mA cm^{-2}. The 30 wt% PSSMA-GF (P30-GF) membrane showed the best long-term cycling performance of full cells.

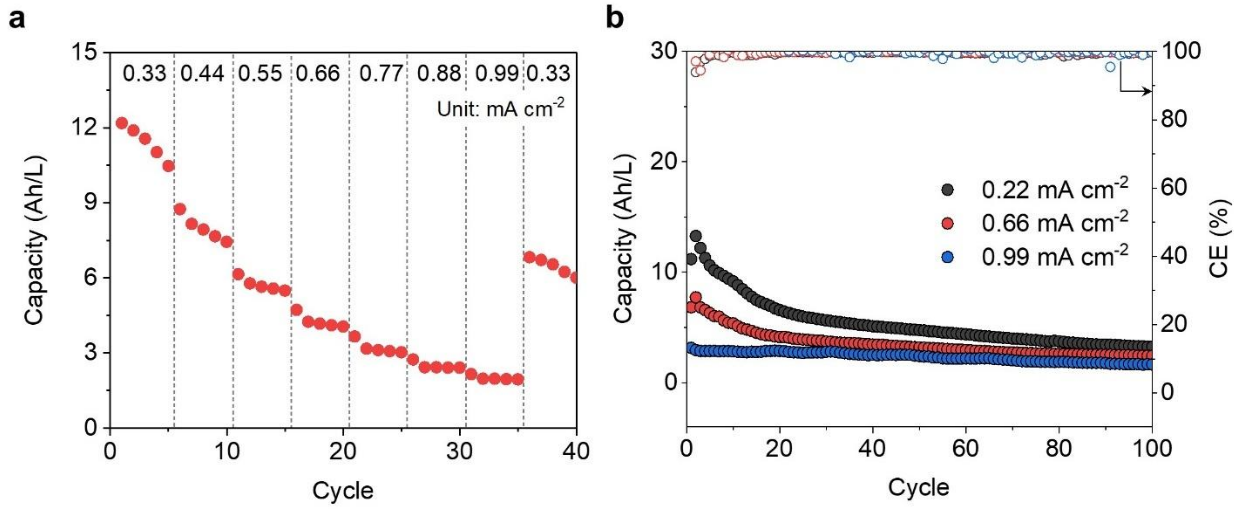

Figure S11. Additional electrochemical evaluation of the PSSMA-GF-based Na||Na_2S_8 full cell at multiple current densities. (a) Rate performance obtained by stepwise increasing the current density from 0.33 to 0.99 mA cm^{-2}, followed by returning to 0.33 mA cm^{-2}. (b) Long-term cycling performance at 0.22, 0.66, and 0.99 mA cm^{-2}.

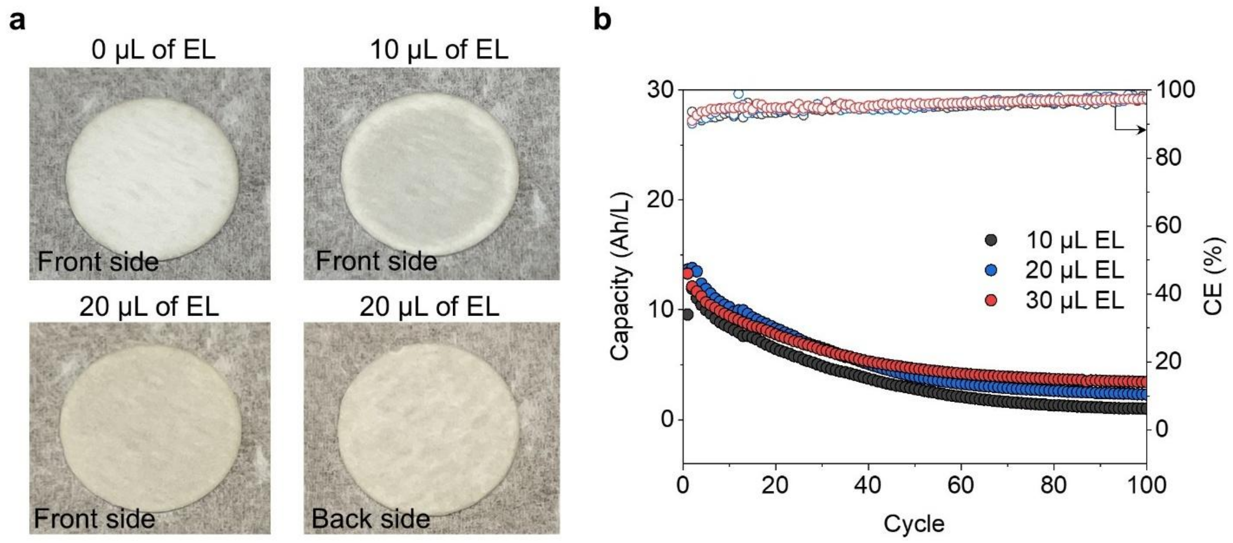

Figure S12. Electrochemical evaluation of the PSSMA-GF-based Na||Na_2S_8 full cell under reduced electrolyte conditions. (a) Photograph of the PSSMA-GF membrane after addition of 10 μL electrolyte, showing incomplete wetting. Front-side and back-side photographs of the PSSMA-GF membrane after addition of 20 μL electrolyte, showing complete wetting across the membrane. (b) Long-term cycling performance and Coulombic efficiency of Na||Na_2S_8 full cells using the PSSMA-GF membrane with electrolyte volumes of 10, 20, and 30 μL.

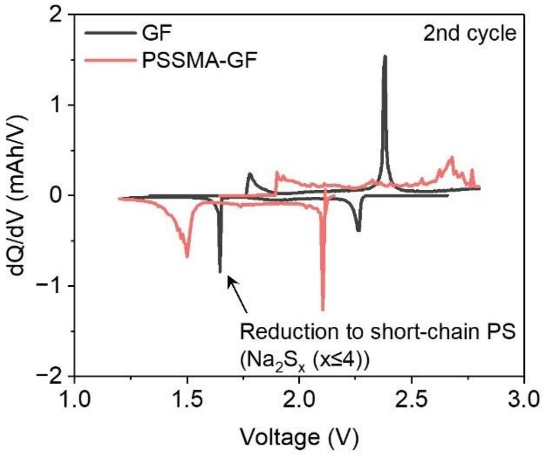

Figure S13. Differential capacity (dQ/dV) profiles of Na||Na_2S_8 full cells with GF and PSSMA-GF membranes at 2nd cycle. The profiles were extracted from the second-cycle galvanostatic charge–discharge curves of the GF-based cell (Figure S3d) and the PSSMA-GF-based cell (Figure S4a). Compared to the GF cell, the PSSMA-GF cell exhibits a reduction peak shifted toward lower potentials, indicating a delayed solid-phase conversion of polysulfides and slower reduction kinetics.

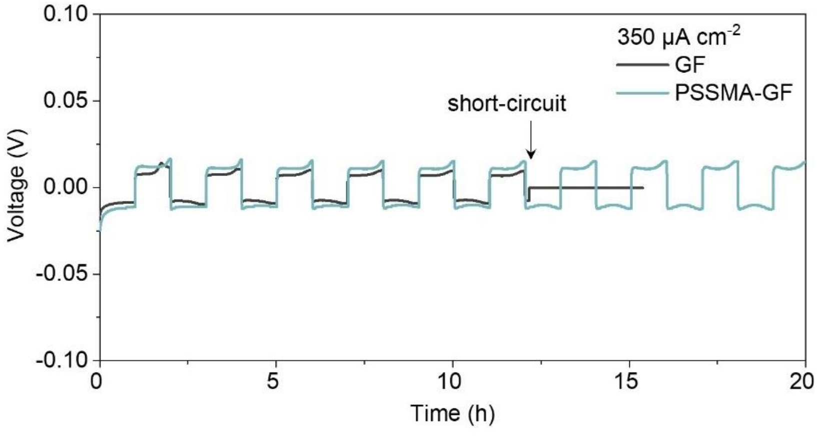

Figure S14. Na|Na symmetric cell tests with GF and PSSMA-GF membranes at a current density of 350 µA cm^{-2}. Although the GF-based cell exhibits a slightly lower initial overpotential, it undergoes rapid short-circuit failure within ~12 h, whereas the PSSMA-GF-based cell maintains stable voltage profiles over prolonged cycling, indicating improved interfacial stability.

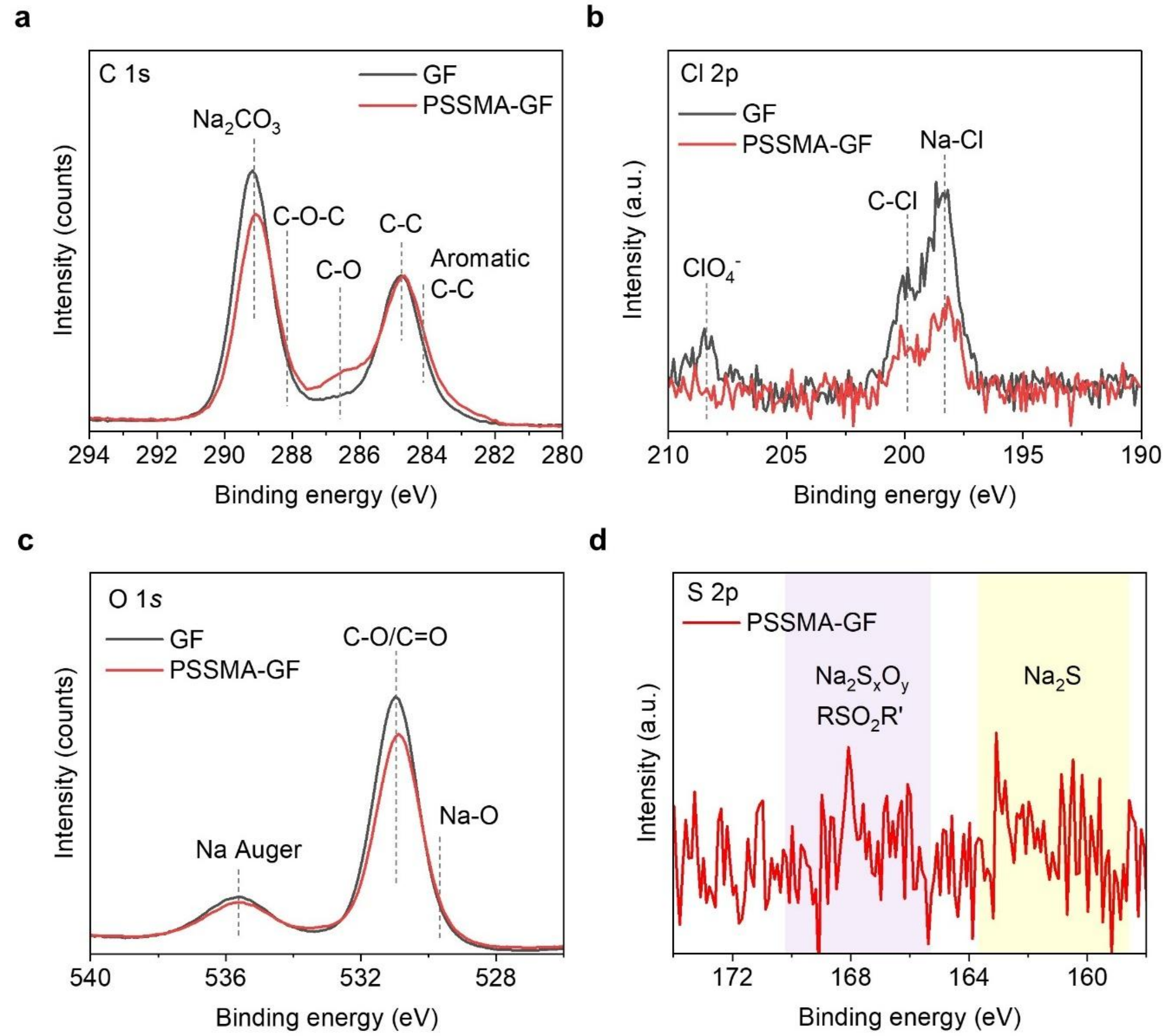

Figure S15. Investigation of the SEI layers formed with GF and PSSMA-GF membranes via XPS analysis. (a) C 1s, (b) Cl 2p, (c) O 1s, and (d) S 2p spectra. Compared to the pristine GF cell, the PSSMA-GF cell exhibits suppressed Cl-related species and enhanced C–O components, indicating reduced $NaClO_4$ decomposition and the formation of a more chemically homogeneous SEI.

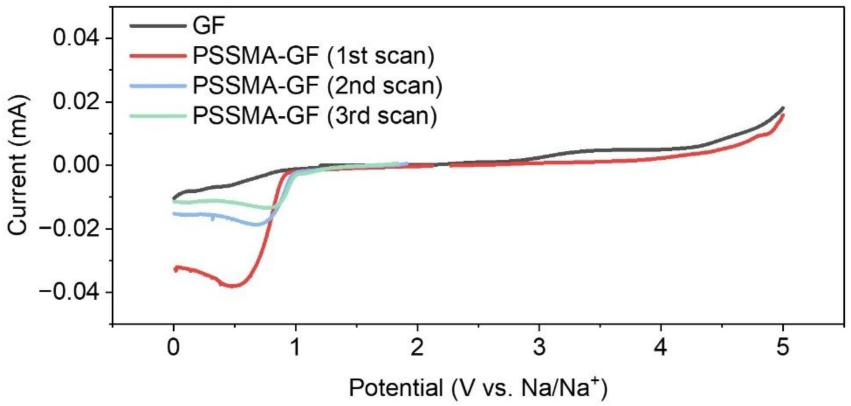

Figure S16. Linear sweep voltammetry profiles of GF and PSSMA-GF membranes. The progressively diminished reduction current over repeated scans suggests that PSSMA participates in the initial SEI formation through reductive decomposition, with limited further electrochemical activity in subsequent cycles.

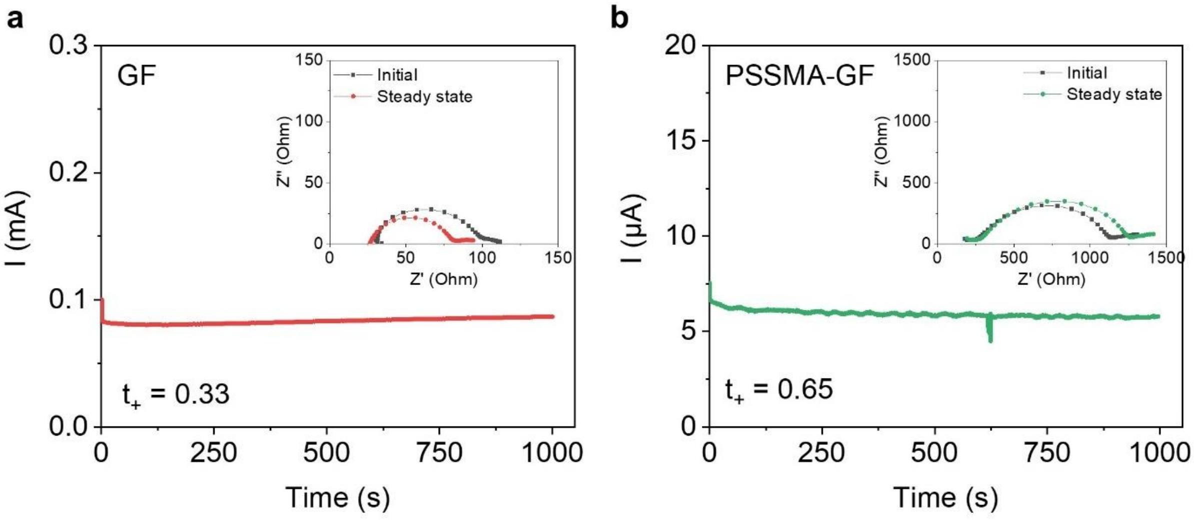

Figure S17. Sodium-ion transference number measurements of pristine GF and PSSMA-GF membranes. Chronoamperometric responses and corresponding impedance spectra before and after polarization for (a) pristine GF and (b) PSSMA-GF. The calculated t_{Na+} values were 0.33 for pristine GF and 0.65 for PSSMA-GF, indicating more Na^+-favored ion transport in the PSSMA-coated membrane.

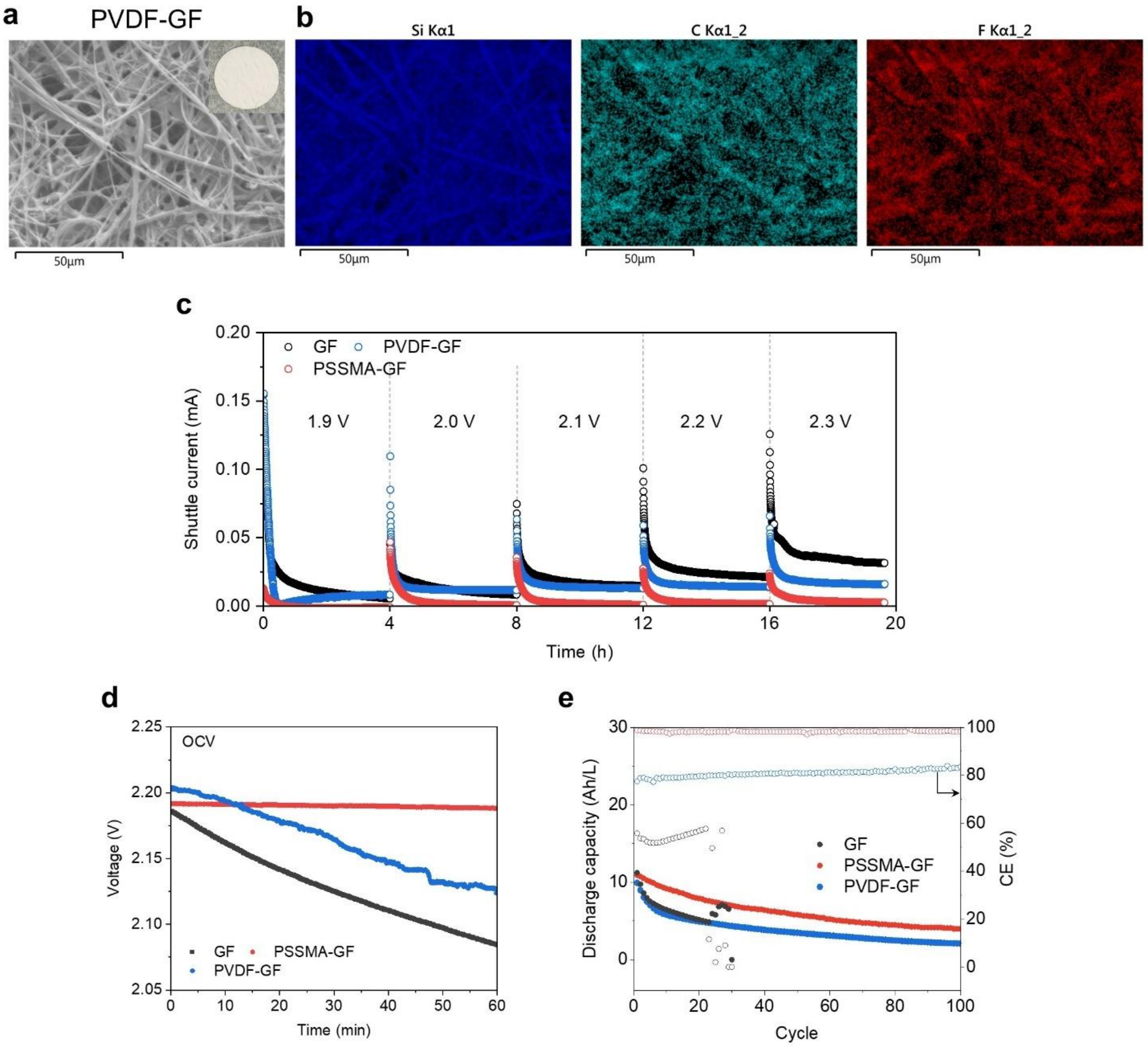

Figure S18. Characterization and electrochemical evaluation of PVDF-GF as a fluorine-rich control membrane. (a) SEM image of the PVDF-GF membrane, with a photograph of the membrane shown in the inset. (b) Corresponding elemental mapping images of Si, C, and F, confirming the presence of the PVDF coating on the glass-fiber framework. (c) Shuttle current measurements of Na||Na_2S_8 full cells using pristine GF, PVDF-GF, and PSSMA-GF membranes at stepwise hold voltages from 1.9 to 2.3 V. (d) Open-circuit voltage evolution of the corresponding full cells. (e) Long-term cycling performance of the corresponding full cells at 0.33 mA cm^{-2}.

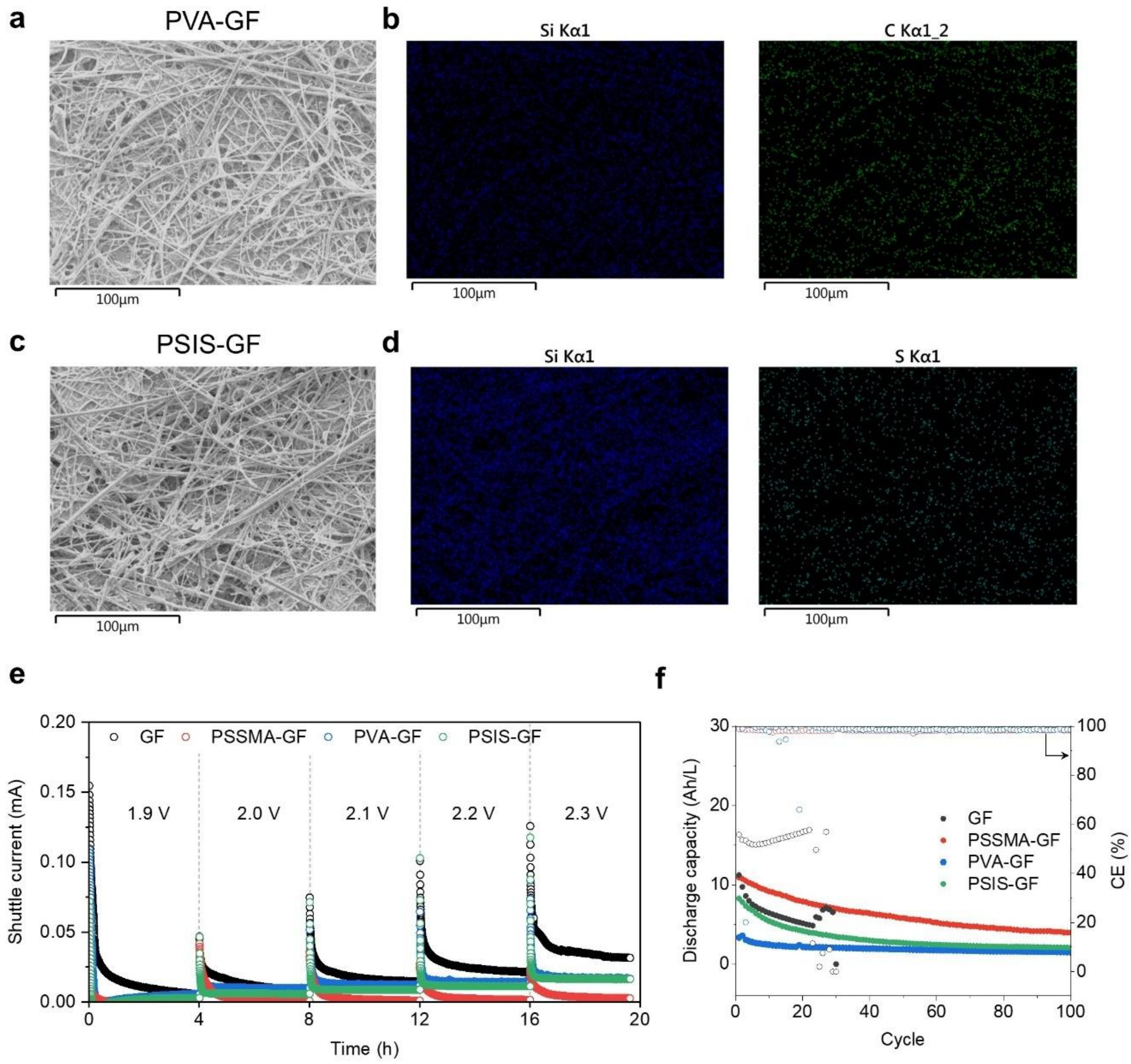

Figure S19. Comparison of additional polymer-coated control membranes for distinguishing fixed-charge effects from simple structural blocking. (a) SEM image of poly(vinyl alcohol)-coated glass fiber (PVA-GF). (b) Corresponding elemental mapping images of Si and C for PVA-GF. (c) SEM image of polystyrene-block-polyisoprene-block-polystyrene-coated glass fiber (PSIS-GF). (d) Corresponding elemental mapping images of PSIS-GF. (e) Shuttle current measurements of Na||Na_2S_8 full cells using pristine GF, PSSMA-GF, PVA-GF, and PSIS-GF membranes at stepwise hold voltages from 1.9 to 2.3 V. (f) Long-term cycling performance of the corresponding full cells at 0.33 mA cm^{-2}.

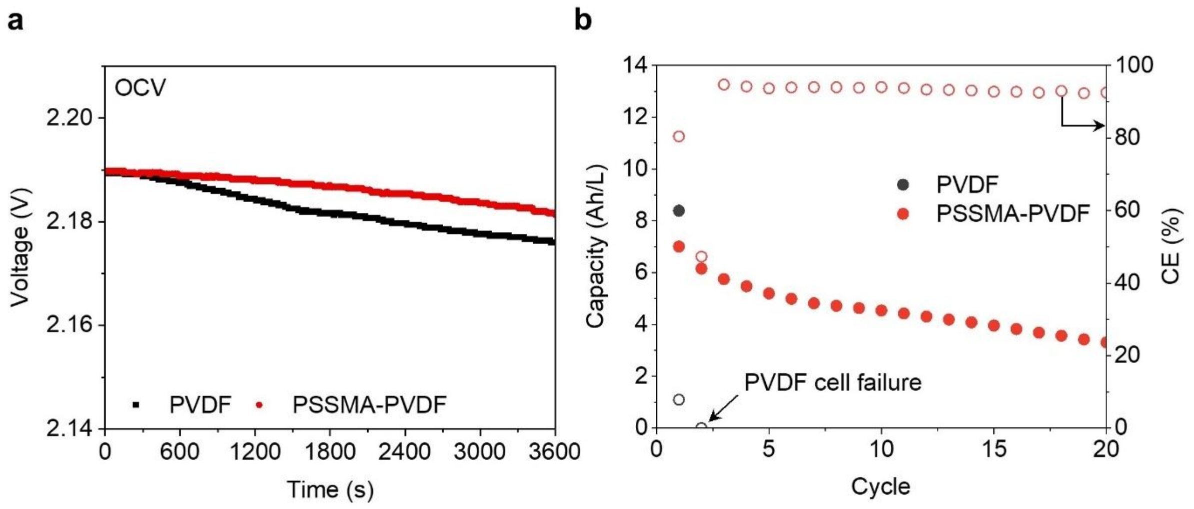

Figure S20. Application of PSSMA coating to PVDF membrane. Na||Na_2S_8 full cells with pristine PVDF and PSSMA-coated PVDF membranes: (a) OCV changes before cycling and (b) short-term cycling performance. The consistent improvement in OCV stability and cycling behavior confirms that the PSSMA coating strategy is broadly applicable to different porous membrane substrates beyond glass fiber.

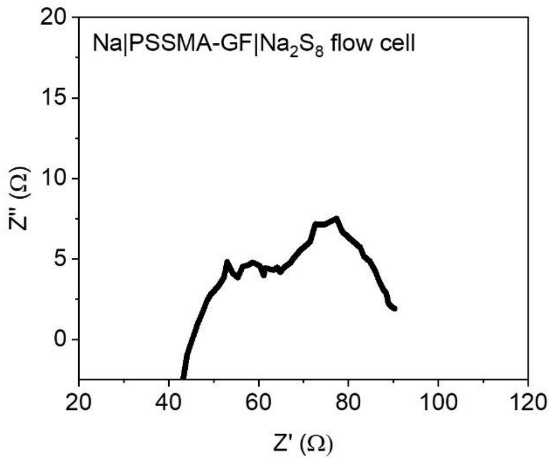

Figure S21. EIS spectrum of the Na|PSSMA-GF|Na_2S_8 flow cell measured before cycling. The flow-cell configuration exhibits a lower overall impedance compared to the coin-cell setup, reflecting improved ionic transport and reduced interfacial resistance under flow conditions.

Table S1. Thickness and calculated apparent density of pristine GF and PSSMA-GF membranes prepared with different PSSMA solution concentrations.

	Thickness (μm)	Apparent density (g/cm^3)
Pristine GF	259	0.298
P5-GF	314	0.268
P10-GF	328	0.281
P15-GF	329	0.301
P20-GF	337	0.325
P30-GF	340	0.386
P40-GF	347	0.463

Table S2. Comparison of reported sodium-polysulfide hybrid redox-flow battery systems and the present work.

	Anode/catholyte/separator	Current density ($mA\ cm^{-2}$)	Capacity ($Ah\ L^{-1}$)	OCV (V)	Energy density ($Wh\ L^{-1}$)
This work	Na metal/0.25 M Na_2S_8 in TEGDME/PSSMA-GF	1.0	4.12	2.04	8.4
Ref. 1	Na metal/0.25 M Na_2S_8 in TEGDME/Nafion 212	0.8	7.28	2.2	16.0
Ref. 2	Na metal/0.1 M Na_2S_8 in TEGDME/Nafion 212	0.5	~4	2.15	~8.6
Ref. 3	Na metal/1 m $NaPF_6$ in diglyme/ Nafion 212	1.0	0.2-0.3	2.28	0.45-0.85

References

[1] ACS Energy Lett. **2024**, 9, 5795-5800.

[2] Adv. Funct. Mater. **2026**, e31310.

[3] Chem. Eng. J. **2026**, 531, 173687.
